# Analysis of the microbial community structure and flavor components succession during salt‐reducing pickling process of *zhacai* (preserved mustard tuber)

**DOI:** 10.1002/fsn3.3297

**Published:** 2023-04-17

**Authors:** Jing Li, Zhifei He, Lixiu Yan, Yunchuan He, Jixia Yang

**Affiliations:** ^1^ College of Food Science Southwest University Chongqing China; ^2^ Chongqing Key Laboratory of Speciality Food Co‐Built by Sichuan and Chongqing Chongqing China; ^3^ Chongqing Academy of Metrology and Quality Inspection Chongqing China; ^4^ Chongqing Fuling Zhacai Group Co. LTD. Er Du Village First Group Chongqing China

**Keywords:** correlation analysis, flavor components, microbial community structure, PacBio SMRT, salt‐reducing pickling, *zhacai*

## Abstract

The salt‐reducing pickling method has been applied to the industrial production of *zhacai*. In order to reveal the succession of the microbial community structure and flavor components during the pickling process, this study used PacBio Sequel to sequence the full length of 16S rRNA (bacteria, 1400 bp) and ITS (fungi, 1200 bp) genes, and detected flavor components simultaneously, including organic acids, volatile flavor components (VFC), monosaccharides, and amino acids. Eleven phyla and 148 genera were identified in the bacterial community, and 2 phyla and 60 genera in the fungal community. During the four stages of pickling, the dominant bacterial genera were *Leuconostoc*, *Lactobacillus*, *Leuconostoc*, and *Lactobacillus*, while the dominant fungal genera were *Aspergillus*, *Kazachstania*, *Debaryomyces*, and *Debaryomyces*, respectively. There were 32 main flavor components (5 organic acids, 19 VFCs, 3 monosaccharides, and 5 amino acids). Correlation heat mapping and bidirectional orthogonal partial least squares (O2PLS) analysis showed that the flora having close relation to flavor components included 14 genera of bacteria (*Leuconostoc*, *Clostridium*, *Devosia*, *Lactococcus*, *Pectobacterium*, *Sphingobacterium*, *Serratia*, *Stenotrophomonas*, *Halanaerobium*, *Tetragenococcus*, *Chromohalobacter*, *Klebsiella*, *Acidovorax*, and *Acinetobacter*) and 3 genera of fungi (*Filobasidium*, *Malassezia*, and *Aspergillus*). This study provides detailed data regarding the microbial community and flavor components during the salt‐reducing pickling process of *zhacai*, which can be used as a reference for the development and improvement of salt‐reducing pickling methods.

## Graphical abstract

This study investigated a whole process of the salt‐reducing pickling of zhacai. The microbial community structure during the four stages was analyzed by using the PacBio Sequel platform to sequence full‐length 16S rRNA and ITS genes, the flavor components were measured (organic acids, VFCs, monosaccharides and amino acids), the correlations between microbial communities and flavor components were analyzed using correlation heat mapping and O2PLS modeling, the core functional flora were inferred through integrated correlation analysis, and the predicted functions of the microbial communities after using PICRUSt2 analysis were highlighted.

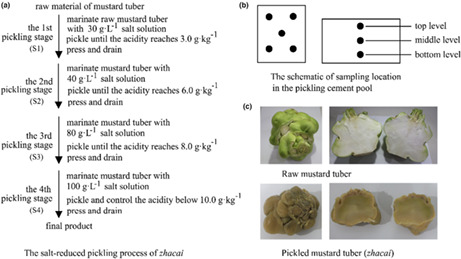

## 1 INTRODUCTION

*Zhacai* is a type of pickle processed using the tuber of the mustard plant *Brassica juncea* as the raw material. *Zhacai* is produced in three major areas in China, Fuling in Chongqing, Yuyao in Zhejiang, and Xiapu in Fujian. Among them, Fuling is well known as the “hometown of *zhacai*,” because it is the origin of *Brassica juncea* varieties as it is located at the intersection of Yangtze River and Wujiang River, and has a unique climate, soil, and other geographical environments which are especially suitable for growing *Brassica juncea* (Fan et al., 2016). In 1898, Qiu Shou'an, a pickle merchant, created the pickling method for this mustard tuber and named it “*zhacai* (pressed vegetable)” because the process includes pressing treatment utilizing wood to expel the brine from the tuber (He & Hou, 2012). As early as 1910–1930, Fuling realized industrialized and large‐scale production of *zhacai* (Yuan, 2008). Presently, the raw mustard tuber output from *Brassica juncea* in Fuling accounts for 70% of production within the entire country, and it is the largest *zhacai* production and export zone.

**FIGURE 1:**
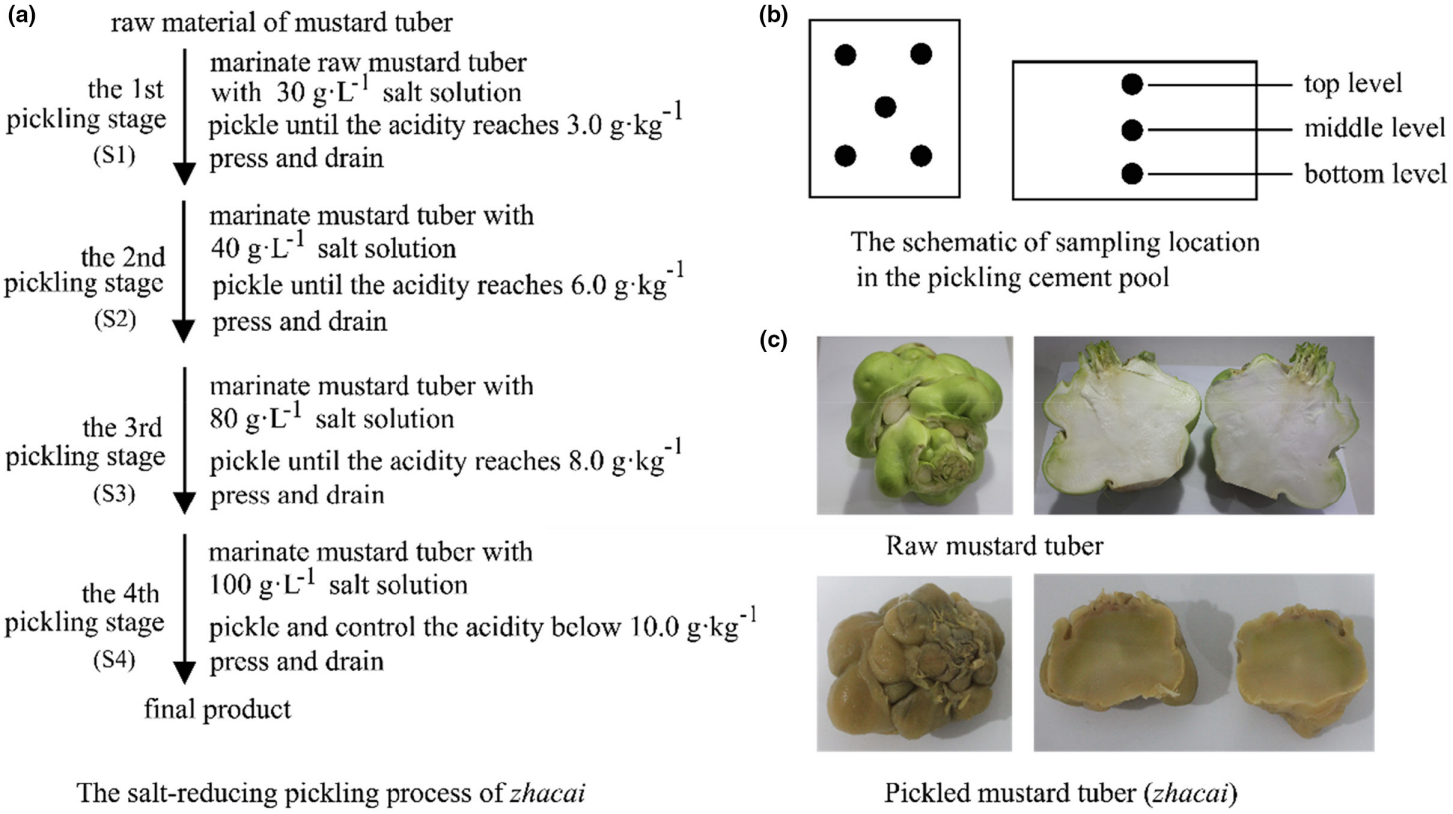
Schematic diagram of (a) salt‐reducing pickling process of *zhacai*, (b) sampling location, and (c) pictures of *zhacai*.

A multistage pickling method is adopted during industrial production of *zhacai*. The traditional method is summarized as “three times pickled and pressed,” as can be seen from the name, the product is pickled three times by immersing the raw tuber in 40, 80, and 140–160 g·L^−1^ (w/v) salt solution in a cement pool for about 7, 20–30 days, and 4–6 months, respectively. At the end of each pickling stage, the tuber is taken out and pressed to remove the brine, and then transferred to the next pickling stage (the tuber reaches its final pickling product at the end of the third stage) (He et al., 2013). We have reported on this traditional pickling method in a previous article (Yang et al., 2021). The other method is a salt‐reducing pickling method which has been developed in recent years. This technique includes four pickling stages, the salt concentration within the brine is about 30, 40, 80, and 100 g·L^−1^, for one to four stages, respectively. The acidity of the tubers at each stage is an important factor in process control. When the acidity in the first three stages reaches 3, 6, and 8 g·kg^−1^, respectively, the pickling is terminated, and the tuber is then pressed and transferred to the next stage. In the fourth stage, the acidity of the final product is controlled and must be less than 10 g·kg^−1^ (as shown in Figure 1a). This new method reduces salt concentrations in the brine, shortens the total pickling time (less than 4 months), and lowers the workload of subsequent desalination treatment; in addition, the flavor and other organoleptic qualities of the *zhacai* are equivalent to that achieved using the traditional method.

The salt‐reducing pickling method has been applied to the industrial production of *zhacai*. However, the succession of the microbial community structure and the formation of flavor components under these processing conditions have not been clarified. The characteristic flavors of *zhacai* include salty, umami, sour, and sweet tastes and unique and rich aroma, which is deeply loved by consumers. *Zhacai* is used as an appetizer and seasoning for Sichuan cuisine. The formation of *zhacai*'s flavor is complex, it includes not only the flavor components of raw mustard tuber but also the flavor components formed by the chemical conversion of endogenous enzymes and microorganisms during the pickling process. The metabolic activities of microorganisms play a key role in the formation of flavor quality. Carbohydrates in the tubers can be degraded into low‐molecular sugars (especially monosaccharides) for use by other microorganisms, or converted into organic acids. Monosaccharides and organic acids produce sweet and sour tastes, respectively. The free amino acids produced by degradation of proteins produce umami, sweet, or bitter taste. Microorganisms can also metabolize and produce volatile flavor components (VFCs), such as esters, aldehydes, and alcohols, that are the main components of *zhacai*'s aroma (Li, 2003). Thus, microorganisms probably contribute to the aroma and umami, sour, and sweet flavors of *zhacai*. *Zhacai* depends mainly on natural microbial fermentation and its long pickling time, and multiple steps can also easily be affected by temperature, environmental, and other factors, making its flavor unstable. Studying and clarifying the evolution process of the microorganisms and flavor components, and exploring the correlations between them, will help to improve flavor stability through process control.

The third‐generation sequencing platform PacBio SMRT (single‐molecule real‐time) has been used to study microbial diversity. It is capable of full‐length sequencing 16S rRNA genes (1400 bp) in bacteria and the ITS gene (1300 bp) in fungi with 99% accuracy (Fichot & Norman, 2013; Roberts et al., 2013; Tedersoo et al., 2020), and improves the accuracy of microbial community structure analysis. This study investigated the entire process of the salt‐reducing pickling method, with tuber and brine samples collected and analyzed at various stages and time points. The full‐length 16S rRNA and ITS genes were sequenced using the PacBio Sequel platform, the flavor components (organic acids, monosaccharides, VFCs, and amino acids) were detected simultaneously, and correlation heat mapping and the bidirectional orthogonal partial least squares (O2PLS) were used to analyze the correlations between the microbial community and flavor components and infer the core functional flora (Huang et al., 2018; Zhao et al., 2020), and PICRUSt2 (Phylogenetic Investigation of Communities by Reconstruction of Unobserved States) was used to predict the function of bacterial and fungal communities based on 16S rRNA and ITS gene sequences, respectively (Douglas et al., 2020). Using these techniques and analyses, it was expected that a reference could be provided to develop and improve the salt‐reducing pickling method.

## 2 MATERIALS AND METHODS

### 2.1 Sample collection

This study investigated the salt‐reducing pickling process for *zhacai*, as described above. The raw mustard tubers were marinated in 30, 40, 80, and 100 g·L^−1^ (w/v) salt solutions, respectively, during the four stages of the pickling process. The acidity of the tubers was controlled at 3.0, 6.0, 8.0, and 10.0 g·kg^−1^ for the four stages, respectively. At the end of the first three stages, the tuber was taken out of the brine, pressed until most water flows out, and then transferred to the next stage. The pickling times of the four stages were 7, 20, 37, and 36 days, respectively (Figure 1a). The color, flavor, and quality of *zhacai* were qualified after being reviewed by enterprise personnel. Samples of brine and tuber were collected on days 4 and 7 of the first stage, days 4, 10, 16, and 20 of the second stage, days 2, 10, 20, and 37 of the third stage, and days 5, 10, 15, 21, 28, and 36 of the fourth stage (as shown in Table 1). To ensure uniformity of sampling, the rectangular cement pickling pool was divided into three layers (i.e., top, middle, and bottom). The four corners and center of each layer were selected as sampling points (5 points per layer, 15 points in total, Figure 1b). Approximately 1 kg tuber and 500 mL of brine were collected at each point. Tuber or brine samples collected at the same time points were pooled and homogenized. The tuber samples were divided into three portions for parallel biochemical analyses. The brine samples were used to analyze microbial community structure.

**TABLE 1:** The information, basic chemical indices, organic acid, and monosaccharide concentrations in *zhacai* samples during the pickling process^1,2^.

Pickling stage	Sample	Pickling days	Titratable acidity^3^ (g·kg^−1^)	NaCl concentration (g·kg^−1^)	Nitrite concentration (mg·kg^−1^)	Organic acid concentration (g·kg^−1^ ${\mathrm{kg}}^{-1}$)					Monosaccharide concentration (μg·kg^−1^)			
						Oxalic acid	Malic acid	Lactic acid	Acetic acid	Succinic acid	Glucose	Fructose	Galactose	Total
Raw material	S0	0	0.77 ± 0.06^q^	5.06 ± 0.17^q^	0.48 ± 0.012^g^	0.55 ± 0.04^c^	0.34 ± 0.05^e^	0.69 ± 0.05^j^	0.39 ± 0.04^i^	2.58 ± 0.11^k^	9.33 ± 0.36^a^	9.26 ± 0.41^a^	0.02 ± 0.01^l^	18.97 ± 0.76^a^
First	S11	4	1.81 ± 0.07^p^	13.85 ± 0.10^p^	0.88 ± 0.011^d^	0.73 ± 0.09^c^	0.53 ± 0.06^d^	3.65 ± 0.15^i^	0.71 ± 0.06^h^	2.84 ± 0.07^j^	8.12 ± 0.23^b^	8.45 ± 0.54^b^	0.05 ± 0.01^l^	16.88 ± 0.79^b^
	S12	7	3.22 ± 0.05^o^	20.75 ± 0.11^o^	0.79 ± 0.046^e^	0.83 ± 0.06^bc^	0.62 ± 0.09^d^	5.41 ± 0.10^h^	1.07 ± 0.08^g^	3.14 ± 0.11^i^	8.62 ± 0.46^b^	7.57 ± 0.56^c^	0.06 ± 0.01^l^	16.50 ± 1.05^b^
Second	S21	4	3.43 ± 0.13^n^	22.41 ± 0.09^n^	0.95 ± 0.046^c^	0.84 ± 0.08^b^	0.68 ± 0.04^d^	5.82 ± 0.09^g^	1.18 ± 0.10^g^	3.49 ± 0.04^h^	7.13 ± 0.17^c^	7.34 ± 0.64^c^	0.14 ± 0.02^l^	14.83 ± 0.63^c^
	S22	10	4.48 ± 0.10^m^	26.64 ± 0.08^m^	1.10 ± 0.048^b^	0.86 ± 0.07^ab^	0.82 ± 0.08^cd^	8.25 ± 0.16^f^	1.88 ± 0.08^f^	4.24 ± 0.20^g^	6.92 ± 0.64^c^	7.16 ± 0.42^cd^	0.38 ± 0.05^k^	14.75 ± 1.15^c^
	S23	16	5.33 ± 0.08^l^	28.16 ± 0.09^l^	0.87 ± 0.048^d^	0.81 ± 0.07^bc^	0.89 ± 0.04^c^	10.22 ± 0.12^e^	2.19 ± 0.12^e^	4.84 ± 0.13^f^	5.89 ± 0.56^d^	7.34 ± 0.47^c^	0.62 ± 0.03^j^	14.43 ± 1.12^c^
	S24	20	6.19 ± 0.05^j^	31.17 ± 0.10^k^	0.96 ± 0.048^c^	0.76 ± 0.05^bc^	0.93 ± 0.03^c^	11.62 ± 0.14^d^	2.02 ± 0.07^ef^	5.26 ± 0.08^e^	5.17 ± 0.23^e^	7.40 ± 0.64^c^	0.81 ± 0.04^i^	14.25 ± 1.01^cd^
Third	S31	2	6.07 ± 0.08^k^	35.81 ± 0.14^j^	0.75 ± 0.038^ef^	0.74 ± 0.05^c^	0.87 ± 0.04^c^	11.84 ± 0.09^d^	1.97 ± 0.04^f^	5.01 ± 0.03^f^	5.59 ± 0.32^de^	6.89 ± 0.67^cd^	1.07 ± 0.12^h^	14.41 ± 1.23^c^
	S32	10	6.79 ± 0.06^i^	49.63 ± 0.18^i^	0.73 ± 0.017^f^	0.78 ± 0.02^bc^	0.94 ± 0.03^c^	12.77 ± 0.26^c^	2.37 ± 0.11^d^	5.73 ± 0.09^d^	5.36 ± 0.52^de^	6.39 ± 0.57^d^	1.29 ± 0.15^g^	13.92 ± 0.67^cd^
	S33	20	7.29 ± 0.04^h^	57.32 ± 0.24^h^	0.85 ± 0.035^d^	0.88 ± 0.04^ab^	1.12 ± 0.07^b^	13.09 ± 0.49^c^	2.91 ± 0.14^c^	6.23 ± 0.08^b^	4.78 ± 0.60^e^	5.79 ± 0.74^de^	1.45 ± 0.12^f^	12.88 ± 1.50^d^
	S34	37	8.07 ± 0.05^g^	66.71 ± 0.16^g^	0.71 ± 0.035^f^	0.93 ± 0.02^a^	1.41 ± 0.16^a^	13.42 ± 0.08^b^	2.75 ± 0.09^c^	6.54 ± 0.14^a^	4.25 ± 0.45^f^	5.88 ± 0.86^de^	1.76 ± 0.13^e^	12.89 ± 1.52^d^
Fourth	S41	5	8.10 ± 0.06^f^	70.49 ± 0.13^f^	0.88 ± 0.012^d^	0.84 ± 0.05^bc^	1.33 ± 0.07^a^	13.70 ± 0.15^b^	2.94 ± 0.10^c^	6.17 ± 0.07^b^	4.03 ± 0.19^f^	5.42 ± 0.64^e^	1.94 ± 0.16^d^	12.47 ± 1.07^de^
	S42	10	8.28 ± 0.03^e^	76.67 ± 0.20^e^	1.26 ± 0.057^a^	0.82 ± 0.02^bc^	1.14 ± 0.04^b^	14.04 ± 0.16^a^	3.22 ± 0.07^b^	5.93 ± 0.12^c^	4.38 ± 0.53^f^	5.28 ± 0.42^e^	2.19 ± 0.14^c^	12.95 ± 1.17^d^
	S43	15	8.47 ± 0.07^d^	81.19 ± 0.12^d^	0.90 ± 0.012^cd^	0.76 ± 0.04^c^	1.28 ± 0.12^ab^	14.20 ± 0.08^a^	3.49 ± 0.20^a^	5.72 ± 0.13^d^	3.23 ± 0.19^g^	5.67 ± 0.33^de^	2.24 ± 0.09^bc^	12.39 ± 0.50^de^
	S44	21	8.69 ± 0.08^c^	83.72 ± 0.14^c^	1.10 ± 0.058^b^	0.73 ± 0.02^c^	1.18 ± 0.11^b^	14.31±0.09 ^a^	3.26 ± 0.12^b^	5.84 ± 0.10^cd^	3.12 ± 0.13^g^	4.92 ± 0.66^ef^	2.49 ± 0.15^a^	11.90 ± 1.03^de^
	S45	28	8.94 ± 0.03^b^	85.47 ± 0.10^b^	1.10 ± 0.050^b^	0.72 ± 0.05^c^	1.09 ± 0.03^bc^	14.23±0.10 ^a^	3.30 ± 0.06^b^	5.63 ± 0.12^d^	3.08 ± 0.18^g^	4.10 ± 0.43^f^	2.46 ± 0.19^ab^	11.09 ± 0.84^e^
	S46	36	9.31 ± 0.06^a^	87.15 ± 0.06^a^	0.94 ± 0.031^cd^	0.70 ± 0.02^c^	0.97 ± 0.09^c^	14.05 ± 0.07^a^	3.17 ± 0.09^b^	5.57 ± 0.13^d^	3.36 ± 0.20^g^	4.35 ± 0.38^f^	2.33 ± 0.19^b^	11.53 ± 0.67^e^

*Note*: The superscript letters which represent significant markers of ANOVA test at the level of *p* < 0.05.

^1^ All data are expressed as: $\;\bar{x}\pm \mathrm{sd},n=3$.

^2^ Significance markers: IBM SPSS Statistics 23 software is used for one‐way ANOVA test, and then is subjected to post hoc comparisons with the Waller–Duncan method and a significance level of 0.05.

^3^ Expressed as g·kg^−1^ after multiplying by the factor (0.09) appropriate to lactic acid.

### 2.2 Detection of flavor components

#### 2.2.1 Biochemical indices and organic acids

Titratable acidity (TA), salt, nitrite, and organic acid concentrations of tuber samples were determined by previously established methods (Yang et al., 2021).

#### 2.2.2 Monosaccharides

The concentration of monosaccharides in *zhacai* was measured by ion chromatography with electrochemical detection (Thermo ICS‐5000+; Thermo Fisher Scientific, USA). Methodological details including standard reagents and standard curves can be found in Appendix S1. *Zhacai* sample (50 mg) was weighed into a 2.0‐mL screw cap glass vial, and 700 μL 80% ethanol was added. The vial was shaken for 2 h at 50°C, then diluted with 700 μL water, centrifuged at 10000 rpm for 3 min, and the supernatant was recovered and adjusted to an appropriate dilution for injection. Ion chromatography used a Dionex CarboPac PA10 column (250 × 4.0 mm, 10 μm) at 30°C and a 20 μL injection volume. Mobile phase A was water and B was 100 mmol·L^−1^ NaOH. The elution gradient (flow rate 0.5 mL·min^−1^) was as follows: 0–30 min mobile phase B increased from 2.5% to 20%, 30.1–45 min held at 40% B, and 45.1–60 min held at 2.5% B.

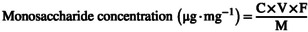

where C is the observed concentration (μg·mL^−1^), V is volume of sample extract (mL), F is dilution factor, and M is mass of sample (mg).

#### 2.2.3 Volatile flavor components (VFCs)

VFCs were determined by solid‐phase microextraction (SPME) and GC–MS (QP2010; Shimadzu, Japan) using a DB‐5MS capillary column (30 m × 0.25 mm, 0.25 μm; Agilent, USA). Homogenized *zhacai* sample (5 g) was placed in a 30‐mL SPME glass vial containing 10 mL of water saturated with salt. Internal standard (10 μL of 100 μg·mL^−1^ ethyl decanoate in absolute ethanol) was added and the vial was sealed. SPME extraction fiber (50/30 μm DVB/CAR/PDMS; Supelco, USA) was inserted into the headspace bottle and VFCs were extracted for 40 min in a 60°C water bath. The fiber was then inserted into the sample inlet (250°C) and desorbed for 5 min. GC conditions were as follows: 40°C (0 min) held for 3 min, 5°C min^−1^ to 100°C then held for 2 min, and 10°C min^−1^ to 250°C then held for 1 min; splitless injection; and helium (purity 99.999%) carrier gas flow 1.0 mL·min^−1^. The mass spectrometer was operated in electron ionization mode (EI) at 250°C and 70 eV, and mass scanning range 35–400 m/z. All analyses were conducted in triplicate. The Retention index value of VFCs was determined using a set of n‐alkanes (C7‐C40, Sigma, USA) standard with the same parameters. Analytes were identified using the NIST 17 library and RI value, and concentrations were calculated using ethyl decanoate as internal standard as follows:

$$M=\frac{\rho \times V\times A_{i}}{A\times m}\times 10^{3}$$

where M is concentration (μg·kg^−1^), ρ is mass concentration of internal standard (μg·mL^−1^), V is volume of internal standard (mL), A_i_ is peak area of the analyte, A is peak area of the internal standard, and m is mass of tuber sample (g). The odor activity value (OAV) of each compound was obtained by dividing the concentration by its corresponding odor threshold. The odor threshold comes from the book compiled by Van Gemert and Nettenbreijer (2011), and the detection thresholds in the latest literature are selected.

#### 2.2.4 Amino acids

The concentrations of 22 amino acids in *zhacai* were determined by ultrahigh‐performance liquid chromatography (ACQUITY, Waters, USA) coupled with a triple quadrupole mass spectrometer (Applied Biosystems, USA) using a C18 column (ACQUITY BEH 2.1 × 100 mm, 1.7 μm; Waters, USA). Method details including standard reagents and standard curves are given in Appendix S2. *Zhacai* sample (50 mg) was weighed into a 2‐mL Eppendorf tube, 400 μL 10% formic acid in methanol‐ddH_2_O (1:1, v/v) was added, vortexed for 30 s, and centrifuged at 12000 rpm for 5 min at 4°C. Ten microliters of supernatant was then diluted with 990 μL 10% formic acid in methanol‐ddH_2_O (1:1, v/v) and vortexed for 30 s. The diluted sample (100 μL) was mixed with 100 μL isotopic internal standard (100 ppb), vortexed for 30 s, and filtered through a 0.22 μm membrane into a microvial.

Chromatography conditions were as follows: injection volume 5 μL, column temperature 40°C, mobile phase A 10% methanol in water (with 0.1% formic acid), mobile phase B 50% methanol in water (with 0.1% formic acid), flow rate 0.3 mL·min^−1^ at 0–8.5 min, and increasing from 0.3 to 0.4 mL·min^−1^ over 8.5–12.5 min. The gradient elution program was as follows: 0–6.5 min mobile phase B increased from 10% to 30%; 6.5–7 min phase B 30% to 100%; 7–8 min phase B held at 100%; 8–8.5 min phase B 100%–10%; and 8.5–12.5 min phase B held at 10%. Mass spectrometry conditions were as follows: electrospray ionization (ESI) source, positive ionization mode, ion source temperature 500°C, ion source voltage 5500 V, collision gas 6 psi, air curtain gas 30 psi, and atomization and auxiliary gases 50 psi. The taste activity value (TAV) of each compound was obtained by dividing the concentration by its corresponding taste threshold.

### 2.3 Microbial community structure analysis

Microbial precipitation particles were collected from 20 mL of *zhacai* brine by centrifuging at 7000 *g* for 15 min. The particles were washed with 1x TE buffer and microbial macrogenomic DNA was extracted using a DNeasy *mericon* food kit (Yang et al., 2021). DNA was sequenced at Shanghai Personal Biotechnology Co. Ltd. (China) using a PacBio Sequel platform. The 16S rRNA gene was sequenced using the primer pair 27F (5'–AGAGTTTGATCMTGGCTCAG‐3′) and 1492R (5'–ACCTTGTTACGACTT–3') with a sequencing length of 1400 bp. The primers for fungi were ITS1F (5'–CTTGGTCATTTAGAGGAAGTAA–3') and LR3 (5'–CCGTGTTTCAAGACGGG–3') with a sequencing length of 1300 bp.

Cutadapt v.2.3 was used to cut the primer fragment (set at −0 to 10) and discard the unmatched primer sequence (Martin, 2011). The “fastq_mergepairs” module of Vsearch (v.2.13.4_linux_x86_64) was used to merge sequences (Rognes et al., 2016). The “fastq_filter” module was used for quality control of the merged sequences and “derep_fulllength” for removing repetitive sequences. The deduplicated sequences were clustered at 98% similarity level using the “cluster_size” module, then chimeras were removed using the “uchime_denovo” module. A Perl pipeline (https://github.com/torognes/vsearch/wiki/VSEARCH‐pipeline) was used to filter chimeras in the sequences following quality control to obtain high‐quality sequences. The high‐quality sequences were clustered at 97% similarity level using the “cluster_size” module, yielding the amplicon sequence variant (ASV) and operational taxonomic unit (OTU) table. Singleton OTUs (i.e., those with an abundance of one in all samples) and their representative sequence ASVs were removed from the OTU table. The high‐quality sequences were processed and analyzed further using QIIME2 (Quantitative Insights Into Microbial Ecology, 2020.8) (Bolyen et al., 2019). The “classify‐sklearn” algorithm with default parameters and the pretrained Naive Bayes classifier were used to perform taxonomic annotation of each ASV sequence (https://github.com/QIIME2/q2‐feature‐classifier) (Bokulich et al., 2018). The ASVs of 16S rRNA genes were annotated using the NCBI database (Stoddard et al., 2015), and ITS genes were annotated using Unite (unite 8.0/its_fungi, release 8.0, http://unite.ut.ee/index.php) (Abarenkov et al., 2010). Thus, taxonomic information on each ASV was obtained and relative abundance tables at each taxonomic level were generated. The α‐diversity indices (Shannon, Simpson, Chao1, and Goods coverage) were calculated by QIIME2. Rarefaction curves, box plots of α‐diversity indices (Chao1, Shannon, Simpson), microbial profiles at phylum and genus level, and principal component analysis (PCA) plots were all constructed using the R package (v.4.1.1). Linear discriminant analysis effect size (LEfSe) was carried out using python's LEfSe package (http://huttenhower.sph.harvard.edu/galaxy/root?tool_id=PICRUSt_normalize) (Segata et al., 2011). PICRUSt2 (https://github.com/picrust/picrust2) was used to predict the functions of bacterial and fungal communities while figures were generated on the R package. Some data analysis was also performed on the website (https://www.genescloud.cn/) of Shanghai Personal Biotechnology Co., Ltd.

### 2.4 Correlation between microbial community structure and flavor components

The bacterial and fungal genera with the highest relative abundance greater than 1% and the average relative abundances greater than 0.1% and major flavor components (organic acid, VFCs, monosaccharides, and amino acids) were selected for correlation analysis. The Hmisc package in R was used to calculate Pearson's correlation coefficients and *p* values among bacteria, fungi, and flavor components and to plot heat maps. O2PLS models were constructed in SIMCA 14.1 to analyze correlations among bacteria, fungi, and flavor components. Genera with variable importance in projection (VIP) predictive values greater than 1.0 and significant positive correlations with more than three flavor components were considered to be the core functional flora.

### 2.5 NCBI sequence number

Sequences of the bacterial 16S rRNA gene and fungal ITS gene were submitted to the Sequence Read Archive (SRA) database of the National Center for Biotechnology Information (NCBI) (https://www.ncbi.nlm.nih.gov/sra), with the registration numbers PRJNA725670 and PRJNA725681, respectively.

## 3 RESULTS

### 3.1 Changes in flavor components during pickling

#### 3.1.1 Chemical indices, organic acids, and monosaccharides

The data of titratable acidity, and the NaCl, nitrite, and organic acid concentrations within the tuber samples during pickling process are shown in Table 1. The titratable acidity was selected as the process control factor, which was controlled at 3.0, 6.0, 8.0, and less than 10.0 g·kg^−1^ for pickling stages 1 to 4, respectively. The mustard tuber was immersed in 30, 40, 80, and 100 g·L^−1^ (w/v) salt solutions during the four stages, respectively. The salt penetrates into each tuber and the tuber is also dehydrated by salt, therefore the salt concentrations within the tubers gradually increase at each stage, in the ranges 13.85–20.75, 22.41–31.17, 35.81–66.71, and 70.49–87.15 g·kg^−1^, respectively. The nitrite concentrations were below 1.30 mg·kg^−1^ during the whole process, which is lower than the 4 mg·kg^−1^ standard applied to “green food” (Ministry of Agriculture and Rural Affairs of the People's Republic of China, 2012). The major organic acids were lactic, succinic, and acetic acids, their concentrations all showed upward trends during the pickling process.

In total, 11 monosaccharides were detected within the *zhacai* samples, and complete data are given in Table S1. Table 1 shows the concentrations of the three major monosaccharides and total monosaccharides. The glucose and fructose concentrations gradually decreased from 9.33 and 9.26 μg·kg^−1^, respectively, in the raw tubers to 3.36 and 4.35 μg·kg^−1^ by the end of the process. The concentrations of galactose gradually increased from 0.02 to 2.33 μg·kg^−1^. The total monosaccharide concentration decreased from 18.97 μg·kg^−1^ in raw tuber to 11.53 μg·kg^−1^. The polysaccharides in the raw tuber can be degraded into monosaccharides, mostly glucose and fructose, and the two sugars are metabolized and utilized by microorganisms. As reflected by the downward trends of the concentration of glucose, fructose, and total monosaccharide, the consumption and utilization of monosaccharides exceed their production.

#### 3.1.2 VFCs

**FIGURE 2:**
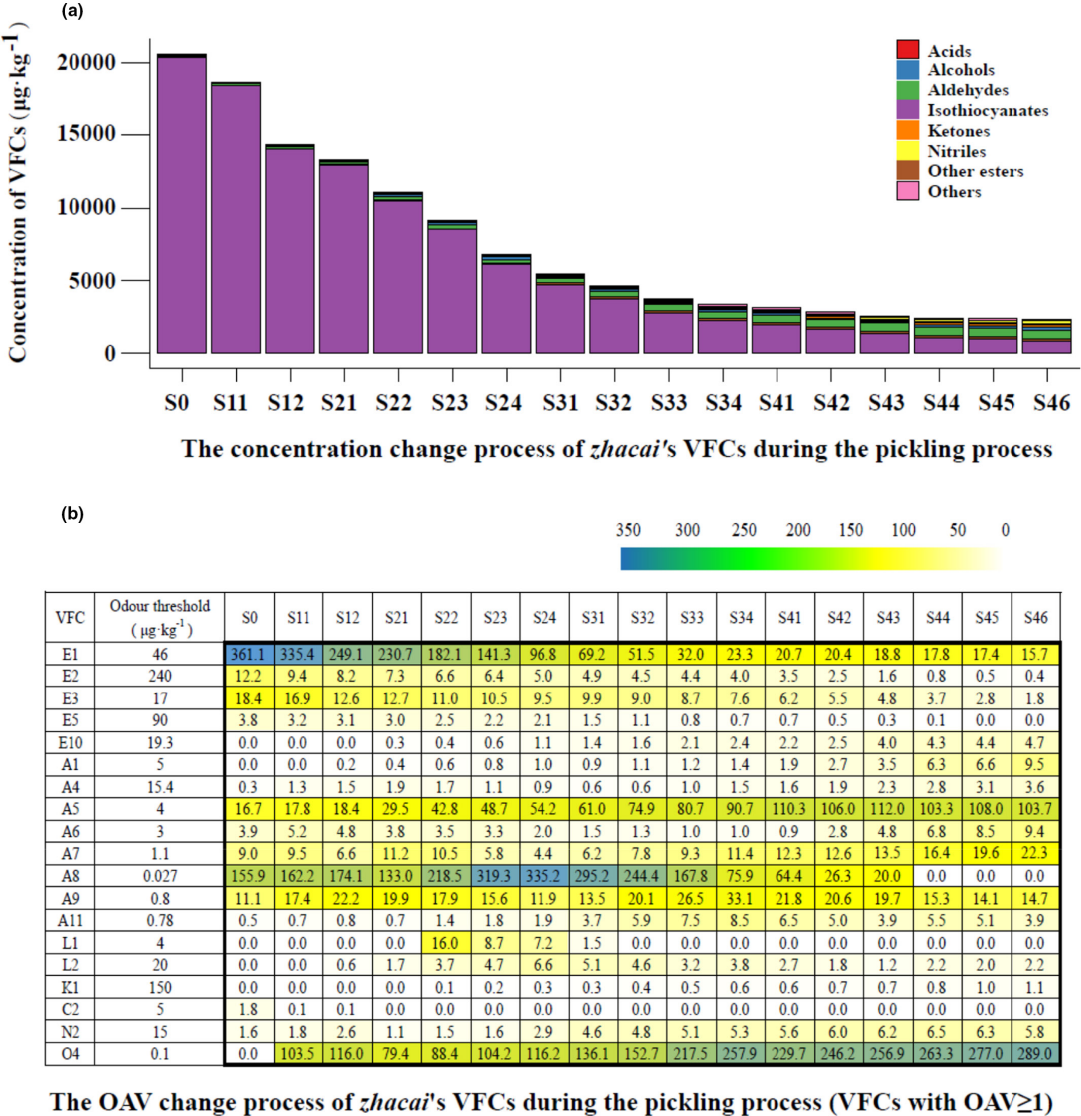
The variation process of *zhacai* VFCs concentration and OAV during pickling process.

In total, 38 VFCs, belonging to seven categories (six categories plus “Others”), were identified in the *zhacai* samples. The complete VFC data are shown in Table S2. Figure 2 shows the concentration changes throughout the process for these seven categories and the VFCs with an OAV greater than 1 (19 in total).

The mustard tuber belongs to *Cruciferae* family and contains characteristic volatile compound isothiocyanates. Eight isothiocyanates were detected from the raw tuber samples in this study, designated as E1‐E8, namely allyl, 2‐phenylethyl, 3‐butenyl, 3‐(methylthio) propyl, butyl, isobutyl, 3‐methylbutyl, and pentyl isothiocyanate. Among them, the concentrations of E1, E2, E3, and E5 were 16612.3, 2919.3, 313.6, and 340.0 μg·kg^−1^, respectively, with an OAV greater than 1, indicating that they were the predominant aroma components of raw tubers, showing a strong pungent aroma. The aldehydes ranked second in the amount of VFCs in the raw mustard tubers. Aldehydes with OAV values greater than 1 included A8 ((E,E)‐2,4‐decadienal), A5 (phenylacetaldehyde), A9 ((E,Z)‐ 2,6‐nonadienal), A7 (nonanal), and A6 ((E)‐2‐octenal), and their aromas are fruity (oily cucumber/melon/citrus), green sweet floral hyacinth, green fatty dry cucumber, waxy aldehydic rose, and fresh cucumber, respectively (all description of aroma were retrieved from http://www.thegoodscentscompany.com/search2.html, the same below). Other components with OAV values greater than 1 were C2 (linolenic acid) and N2 (benzenepropanenitrile), and their aromas are faint fatty and powerful nasturtium, respectively.

During the pickling process, the concentrations of isothiocyanates decreased significantly, and the strong pungent aroma became lighter. The other esters (E9–E13), aldehydes, alcohols, ketones, acids, nitriles, and other components increased from 0, 115.0, 0, 6.9, 29.3, 49.9, and 29.4 μg·kg^−1^, to 135.5, 614.2, 167.6, 197.3, 73.2, 165.9, and 77.4 μg·kg^−1^, respectively. This indicated that significant amounts of these compounds were produced during the pickling process, which changed the composition of aroma components of the tuber samples and made the aroma softer and richer.

Although the concentration of isothiocyanates present decreased significantly, it was still the category with the highest concentration of VFCs in the final pickled product (S46), and the OAVs of E1 and E3 were greater than 1. The other ester component E10 (octanoic acid ethyl ester) also showed an OAV greater than 1, its aromas have been described as fruity wine, waxy, and sweet apricot. The second category was still the aldehydes, and there were seven aldehydes with an OAV exceeding 1, namely A5, A7, A9, A1 (hexanal), A6, A11 (2‐undecenal), and A4 ((E,E)‐2,4‐heptadienal). The aromas of A1, A11, and A4 have been described as fresh green fatty aldehydic grass, oil and green grass, and fatty green oily aldehydic vegetable, respectively. The concentration of O4 (dimethyl trisulfide) reached 28.90 μg·kg^−1^, but its odor threshold was as low as 0.1 μg·kg^−1^ (Van Gemert & Nettenbreijer, 2011), so its OAV value was the highest (289) among all of the VFCs in the final product (S46). Its aroma is described as sulfurous cooked onion savory meaty. Other components with OAV values above 1 included L2 ((E)‐2‐octen‐1‐ol), K1 (3,5‐octadien‐2‐one), and N2, with the aromas of L2 and K1 described as green citrus vegetable fatty and fruity fatty mushroom, respectively. They probably make a great contribution to the aroma of the final pickling product (*zhacai*).

#### 3.1.3 Amino acids

**FIGURE 3:**
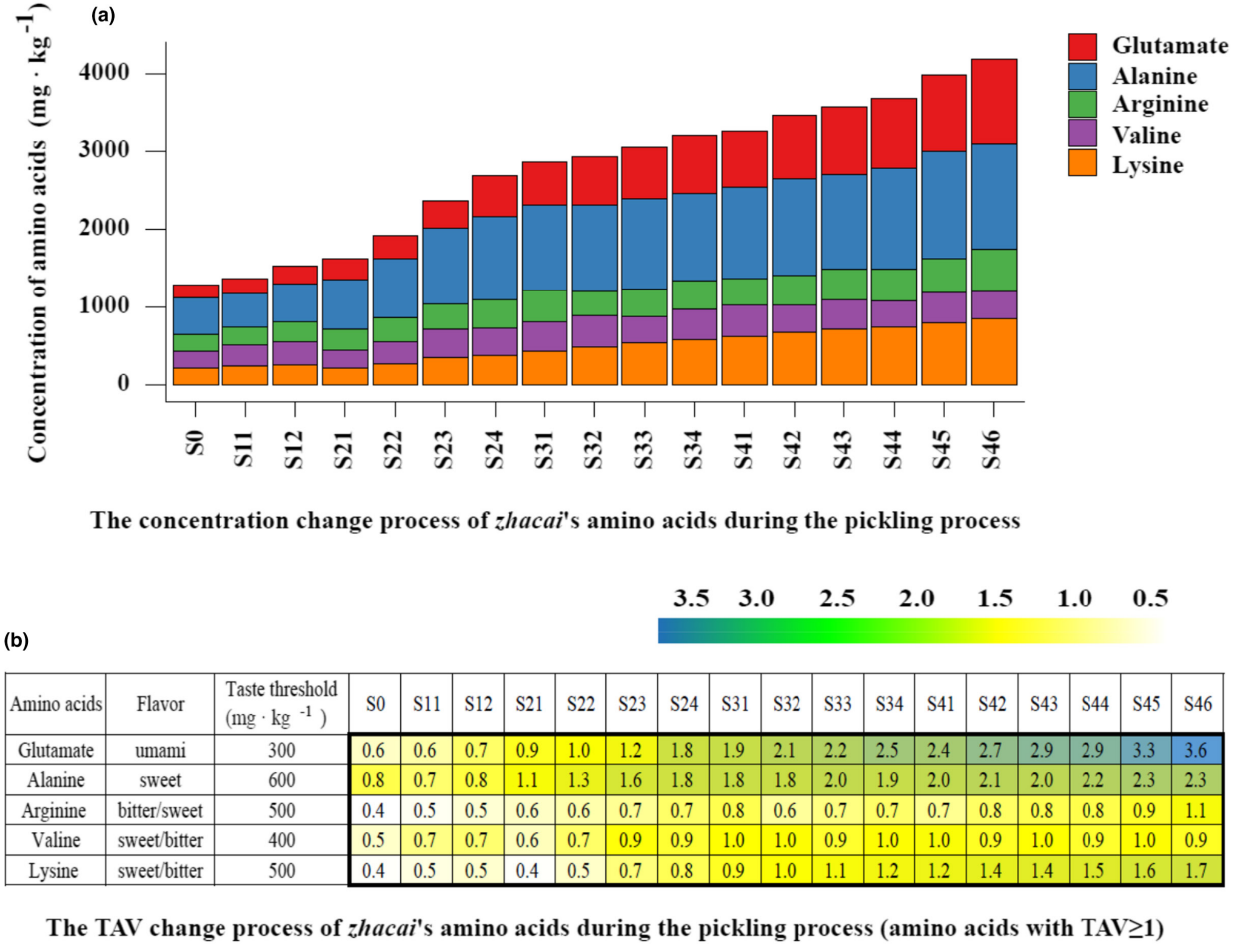
The variation process of *zhacai* amino acids concentration and TAV during pickling process.

In total, 21 amino acids were detected within the *zhacai* samples. The complete dataset is shown in Table S3, while Figure 3 shows the major amino acids. The taste threshold value (TAV) refers to the literature (Zhuang et al., 2016). The TAV of all amino acids in the raw tuber was below 1, so their content probably is not high enough to produce a taste.

During the pickling process, the concentration of five major flavoring amino acids, glutamate, alanine, arginine, valine, and lysine, increased from 164.8, 477.4, 214.1, 215.8, and 218.6 mg·kg ^−1^ in raw tuber to 1091.9, 1360.1, 539.9, 351.1, and 849.9 mg·kg ^−1^ by the end of the process. In the final pickling sample (S46), the TAV of glutamate, alanine, lysine, and arginine reached 3.6, 2.3, 1.7, and 1.1, respectively. These data indicate that a large amount of flavoring amino acids are produced in the pickling process, which probably contributes to *zhacai*'s overall flavor. Glutamate is an important umami taste amino acid. Alanine elicits a sweet and umami taste and is widely used as a flavor enhancer in soy sauce, fish sauce, and other condiments, to make the taste richer and more umami and intense (Kawai et al., 2002; Lioe et al., 2004; Wakinaka et al., 2019). These two amino acids may contribute toward the strong umami and sweet aftertaste of *zhacai*. The lysine and arginine present sweet/bitter and bitter/sweet tastes, respectively, which probably also contribute to flavor. The concentration of γ‐aminobutyric acid (GABA) in the raw tuber was as high as 1350.7 mg·kg ^−1^, and its concentration showed an increasing trend during pickling, reaching 2767.5 mg·kg ^−1^ in the final pickled sample (S46). γ‐Aminobutyric acid is not a flavoring amino acid, but it has important physiological functions.

### 3.2 Microbial community structure succession during pickling

Tables S4 and S5 show the PacBio Sequel sequencing data and α‐diversity indices of the bacteria and fungi, and Tables S6 and S7 show the OTU table of bacteria and fungi, respectively. In the bacterial community, the average number of non‐singletons per sample was 4989.47 ± 1087.24 and the 95% confidence interval of the sequence length was 1478.39–1479.09 bp. Eleven phyla and 148 genera were identified in total. In the fungal community, the average number of nonsingletons per sample was 5811.65 ± 2104.19 and the 95% confidence interval of the sequence length was 1228.75–1230.20 bp. Two phyla and 60 genera were identified overall.

Figure S1 shows the rarefaction curves for the bacteria and fungi. As sequencing depth increased, the curves plateaued, indicating that the vast majority of species had been identified. The “Goods coverage” index for bacteria and fungi (Tables S4 and S5) was always greater than 0.97, which also indicated that most species had been identified and that the sequencing depth was sufficient. Figures S2a,b show the diversity indices and PCA plots for the bacterial and fungal communities, respectively. Paired *t*‐tests showed significant differences in the bacterial Chao 1 indexes between stages 3 and 4 (*p* = .0024, <.01), and in the bacterial Shannon index between stages 1 and 4 (*p* = .034, <.05). Significant differences were also detected in the fungal Shannon index between stages 1 and 3 (*p* = .036, <.05) and between stages 1 and 4 (*p* = .0082, <.01), and in the Simpson index between stages 1 and 3 (*p* = .029, <.05) and stages 1 and 4 (*p* = .0027, <.01). This suggests that the significantly reduced bacterial and fungal diversity observed in the fourth stage of processing may have been due to the high salt concentration (100 g·L^−1^). Figure S2c displays the PCA plots from both communities at a genus level. Stages 1 and 4 samples exhibited distinct separation but the other stages were not clearly clustered.

**FIGURE 4:**
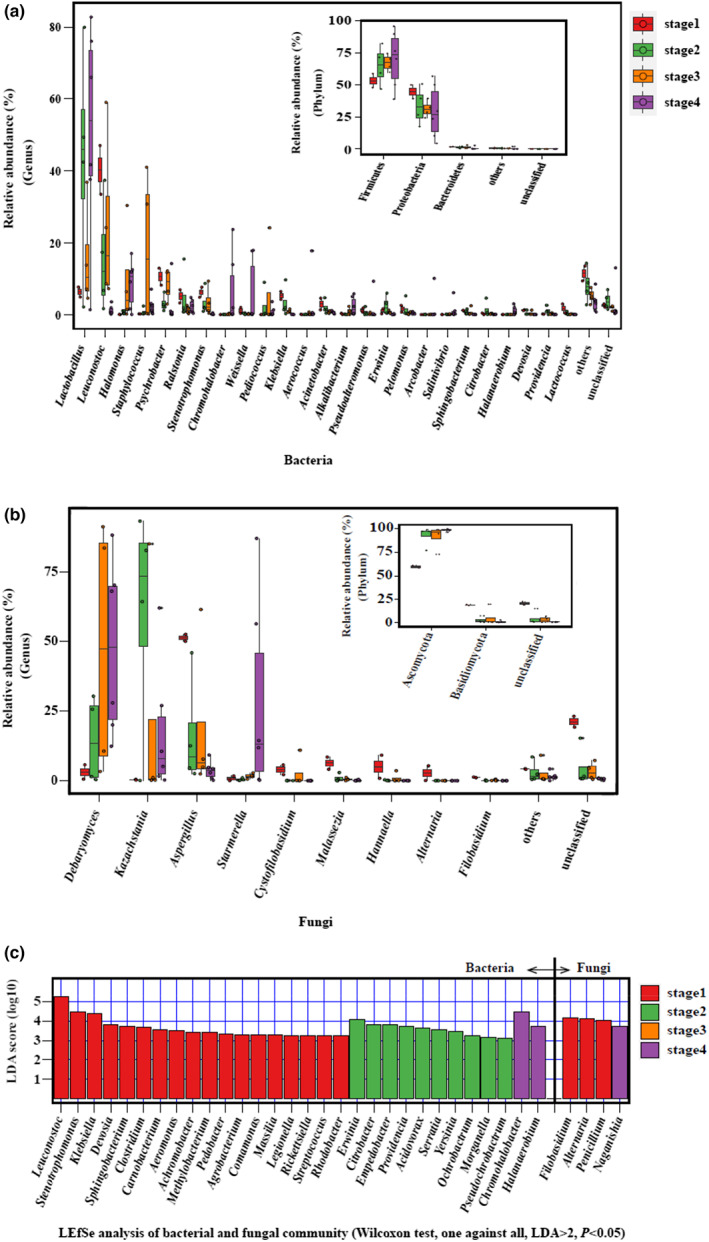
Succession pattern of bacterial (a) and fungal (b) community structure during the *zhacai* pickling process, and (c) LEfSe difference analysis chart.

The succession pattern of bacterial community structure is shown in Figure 4a. In the first stage, the dominant phyla (average relative abundance >1%) included Firmicutes (average relative abundance 53.20%), Proteobacteria (44.38%), and Bacteroidetes (1.67%). The dominant genera were *Leuconostoc* (40.26%), *Psychrobacter* (10.50%), *Lactobacillus* (6.23%), *Stenotrophomonas* (6.19%), *Klebsiella* (5.29%), *Ralstonia* (5.04%), *Acinetobacter* (2.91%), *Lactococcus* (1.68%), *Pelomonas* (1.61%), *Pseudoalteromonas* (1.26%), *Devosia* (1.19%), *Weissella* (1.14%), and *Sphingobacterium* (1.09%). In the second stage, the abundance of Firmicutes increased (increasing to 64.86%), Proteobacteria decreased (down to 33.38%), and Bacteroidetes maintained its previous level (1.23%). At the genus level, *Lactobacillus* proliferated rapidly, becoming the dominant genus (abundance 43.47%) and the abundance of *Leuconostoc* decreased to 15.78%, ranking second. The other major genera included *Ralstonia* (4.72%), *Stenotrophomonas* (3.31%), *Klebsiella* (3.29%), *Psychrobacter* (3.04%), *Erwinia* (2.46%), *Pediococcus* (2.32%), *Acinetobacter* (1.93%), *Pelomonas* (1.53%), *Citrobacter* (1.32%), and *Providencia* (1.05%). In the third stage, Firmicutes (67.21%) and Proteobacteria (31.05%) were still the dominant phyla, followed by Bacteroidetes (1.26%). The dominant genera changed significantly as salt concentrations increased to 80 g·L^−1^. *Leuconostoc* became the dominant genus (24.75%) followed by salt‐tolerant *Staphylococcus* (18.06%), in addition to *Lactobacillus* (15.55%), *Halomonas* (9.57%), *Psychrobacter* (8.29%), *Pediococcus* (6.05%), *Stenotrophomonas* (3.49%), and *Ralstonia* (1.30%). In the fourth stage, the salt concentration increased further (100 g·L^−1^) and Firmicutes (70.05%) and Proteobacteria (28.99%) remained the dominant phyla. *Lactobacillus* (50.94%) became the dominant genus again, and the salt‐tolerant genera proliferated, which included *Halomonas* (8.67%), *Chromohalobacter* (6.63%), *Staphylococcus* (2.38%), *Alkalibacterium* (2.26%), *Pseudoalteromonas* (1.61%), *Salinivibrio* (1.26%), and *Halanaerobium* (1.02%). The other genera present included *Weissella* (6.04%), *Aerococcus* (3.12%), *Psychrobacter* (2.63%), *Ralstonia* (2.16%), *Arcobacter* (1.69%), and *Leuconostoc* (1.19%).

The succession pattern of fungal community structure is shown in Figure 4b. In the first stage, the dominant phyla (average relative abundance >1%) included Ascomycota (average relative abundance 59.75%) and Basidiomycota (19.20%). The dominant genera were *Aspergillus* (51.24%), *Malassezia* (6.28%), *Hannaella* (5.01%), *Cystofilobasidium* (3.91%), *Debaryomyces* (3.12%), *Alternaria* (2.70%), and *Filobasidium* (1.19%). In the second stage, the abundance of Ascomycota increased sharply (92.66%), whereas Basidiomycota levels decreased (2.75%). At the genus level, *Kazachstania* proliferated rapidly and became the dominant genus (60.12%), followed by *Aspergillus* (16.37%) and then *Debaryomyces* (14.45%). In the third stage, Ascomycota (91.33%) remained the dominant phylum, followed by Basidiomycota (5.38%). At the genus level, *Debaryomyces* proliferated rapidly and became the dominant genus (47.19%), followed by *Kazachstania* (21.64%), *Aspergillus* (19.15%), *Cystofilobasidium* (2.76%), and *Starmerella* (1.66%). In the fourth stage, Ascomycota (98.37%) was still the dominant phylum and *Debaryomyces* remained the dominant genus (47.82%), followed by *Kazachstania* (17.76%), *Aspergillus* (3.70%), and *Starmerella* (28.37%).

Linear discriminant analysis effect size (LEfSe) can be used to identify species with significant differences and a sense of biomarker between groups. Figure 4c shows the LEfSe analysis of the bacterial and fungal communities at the genus level. There were 18, 10, and 2 genera in bacterial community, which were enriched in stages 1, 2, and 4 respectively, and 3 and 1 genera in the fungal community were enriched in stages 1 and 4, respectively. These genera can be taken as biomarker (the most representative genus that can be distinguished from that in other stages) in their respective stages.

### 3.3 Correlation between microbial community structure and flavor components

During the four pickling stages, there were 38 bacterial genera and 12 fungal genera with the highest relative abundance measured at greater than 1% and their average relative abundances calculated at greater than 0.1%, and 32 main flavor components (5 organic acids, 19 VFCs, 3 monosaccharides, and 5 amino acids). These data were subjected to correlation analysis.

#### 3.3.1 Correlation analysis by heat mapping

**FIGURE 5:**
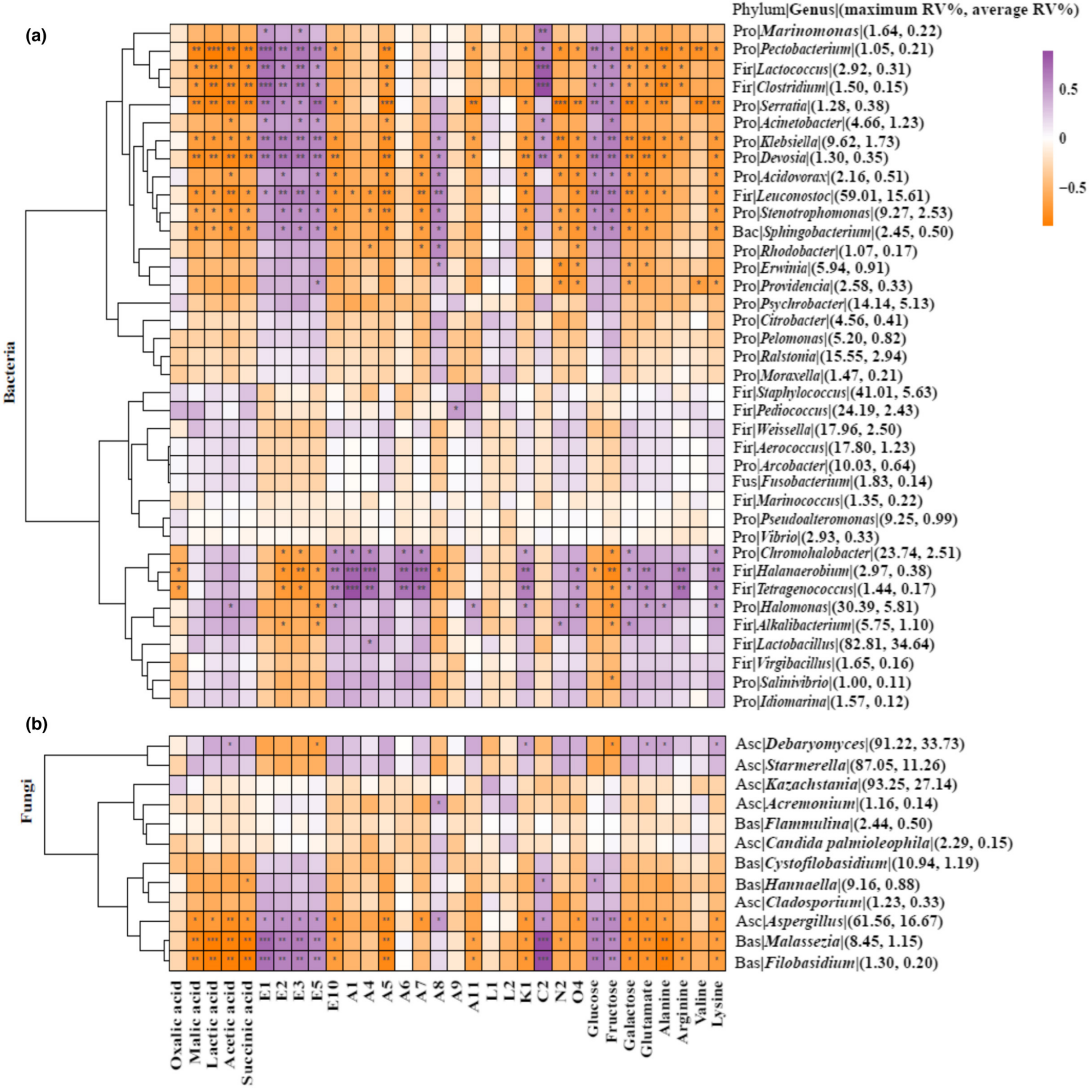
Pearson correlation heat map of bacteria, fungi, and the major *zhacai* flavor components.

Figure 5 shows the Pearson correlation heat maps of the bacteria and fungi against the main flavor components. Mostly significant negative correlations were observed between the five organic acids and the genera of the bacteria and fungi.

The isothiocyanates (E1, E2, E3, and E5) showed a significantly positive correlation with bacterial genera *Marinomonas*, *Pectobacterium*, *Lactococcus*, *Clostridium*, *Serratia*, *Acinetobacter*, *Klebsiella*, *Devosia*, *Acidovorax*, *Leuconostoc*, *Stenotrophomonas*, and *Sphingobacterium*, and was also positively correlated with fungal genera *Aspergillus*, *Malassezia*, and *Filobasidium*. Ester E10 was significantly positively correlated with *Chromohalobacter*, *Halanaerobium*, *Tetragenococcus*, and *Halomonas*.

Some aldehydes (A1, A4, A6, and A7) were positively correlated with *Chromohalobacter*, *Halanaerobium*, and *Tetragenococcus*. A8 was significantly positively correlated with the bacterial genera *Klebsiella*, *Devosia*, *Acidovorax*, *Leuconostoc*, *Stenotrophomonas*, *Sphingobacterium*, *Rhodobacter*, and *Erwinia*, and with the fungal genera *Acremonium* and *Aspergillus*.

Monosaccharides (glucose and fructose) were significantly positively correlated with the bacterial genera *Pectobacterium*, *Lactococcus*, *Clostridium*, *Serratia*, *Acinetobacter*, *Klebsiella*, *Devosia*, *Acidovorax*, *Leuconostoc*, *Stenotrophomonas*, and *Sphingobacterium*, and positively correlated with the fungal genera *Aspergillus*, *Malassezia*, and *Filobasidium*. Galactose was significantly positively correlated with the bacterial genera *Chromohalobacter*, *Halanaerobium*, *Tetragenococcus*, and *Alkalibacterium*.

The five amino acids were significantly positively correlated with the bacterial genera *Chromohalobacter*, *Halanaerobium*, *Tetragenococcus*, *Halomonas*, and *Alkalibacterium*, and with the fungi genus *Debaryomyces*.

#### 3.3.2 Correlation analysis by O2PLS modeling

**FIGURE 6:**
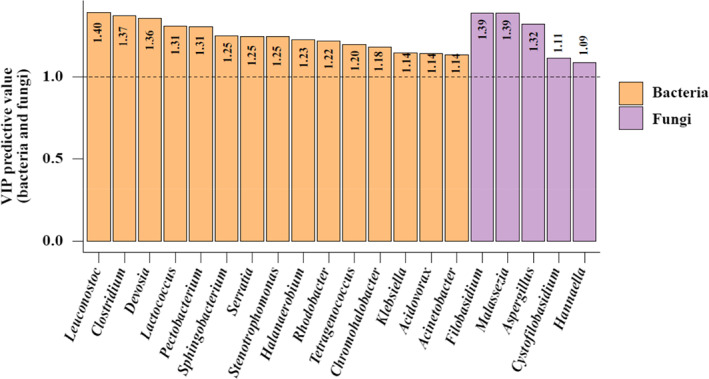
The genera with VIP predictive value greater than 1.0 in bacterial and fungal communities by O2PLS model analysis.

An O2PLS model was constructed using SIMCA 14.1, with X representing the bacterial and fungal data (50 genera in total) and Y representing the flavor component data. The parameters R^2^X (cum), R^2^Y (cum), and Q^2^ (cum) used for evaluating the validity of the O2PLS model were 0.996, 0.966, and 0.538, respectively. All were greater than 0.5, and the first two were closer to 1, which indicated that the fitting effect of the model was good, its prediction was statistically significant (Li et al., 2019; Wang, 2016; Zheng et al., 2018), and the bacteria and fungi data helped explain the flavor components to a certain extent. Figure 6 shows the bacterial and fungal genera with VIP predictive values greater than 1.0, which probably have close correlations with the flavor components.

#### 3.3.3 Integrated correlation analysis

Based on the microbial community structure analysis, correlation heat mapping and O2PLS modeling, 14 genera of bacteria with VIP predictive values greater than 1.0 and significant positive correlations with at least three flavor components were identified: *Leuconostoc*, *Clostridium*, *Devosia*, *Lactococcus*, *Pectobacterium*, *Sphingobacterium*, *Serratia*, *Stenotrophomonas*, *Halanaerobium*, *Tetragenococcus*, *Chromohalobacter*, *Klebsiella*, *Acidovorax*, and *Acinetobacter*. Three genera of fungi also met the above criteria, *Filobasidium*, *Malassezia*, and *Aspergillus*. These 17 genera are inferred to be core functional flora in the fermentation process and are probably closely related to the formation of major flavor components.

### 3.4 Prediction of bacterial and fungal community function by PICRUSt2

**FIGURE 7:**
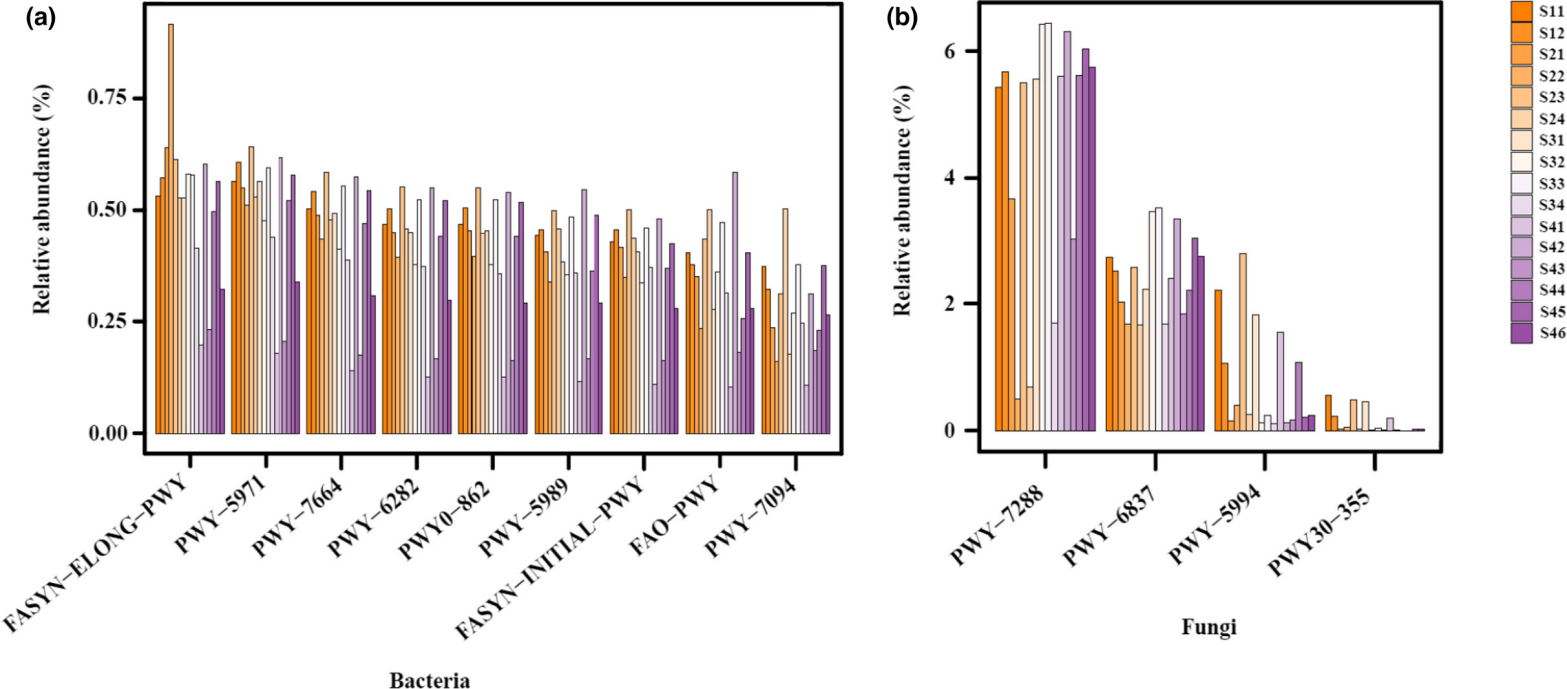
The KEGG pathway involved with the fatty acid metabolism predicted by PICRUSt2 in bacterial (a) and fungal (b) communities.

According to the KEGG pathway prediction by PICRUSt2, both bacterial and fungal communities included several pathways relating to fatty acid metabolism (Figure 7).

## 4 DISCUSSION

This study investigated the whole process of the salt‐reducing pickling of *zhacai*. The microbial community structure during the four stages was analyzed using the PacBio Sequel platform to sequence full‐length 16S rRNA and ITS genes, the flavor components were measured (organic acids, VFCs, monosaccharides, and amino acids), the correlations between microbial communities and flavor components were analyzed using correlation heat mapping and O2PLS modeling, the core functional flora was inferred through integrated correlation analysis, and the predicted functions of the microbial communities after using PICRUSt2 analysis were highlighted.

The traditional pickling process for *zhacai* includes pickling the tubers three times in a high concentration of salt solution (brine). On the third occasion, the salt concentration is the highest at around 140–160 g·L^−1^ and the immersion time is longer at around 4–6 months. The high salt concentrations inhibit the lactic acid fermentation of the microorganisms and make the acidity increase slowly, ensuring that the microorganisms have a longer time to produce the various flavor components (He et al., 2013). The salt‐reducing pickling process investigated in this study includes four pickling stages. Compared with the traditional one, one pickling and one pressing operation are added in this newer technique, and the salt concentrations of the brine in each stage are lower, typically at 80 and 100 g·L^−1^ in the third and fourth stages, respectively, than utilized in the traditional method. This also means that the acid‐producing metabolism stages for the microorganisms are relatively fast, and the pickling time is shortened (about 100 days in total), yet the flavor of the final pickling product (*zhacai*) is equivalent to that of the traditional method. The pressing treatment plays an important role in the salt‐reducing method. It not only helps to expel water from inside tuber and promote the penetration of salt into tuber but also facilitates the outflow of various components inside tuber for microbial metabolism and utilization, accelerating the transformation reactions of chemical components. Thus, although the pickling time is shortened, microorganisms still can complete metabolic transformation of various components and produce large amount of flavor components. Therefore, the addition of pressing operation is necessary for the salt‐reducing pickling process.

This study reveals that the most dominant genera of bacteria in the four pickling stages are *Leuconostoc*, *Lactobacillus*, *Leuconostoc*, and *Lactobacillus*, and the dominant fungal genera were *Aspergillus*, *Kazachstania*, *Debaryomyces*, and *Debaryomyces*, respectively, whereas in all three pickling stages of traditional method, the most dominant bacterial genus is *Lactobacillus*, and dominant fungal genus cannot be determined according to a previous report (Yang et al., 2021). The genus *Leuconostoc* belongs to lactic acid bacteria, its cells are ellipsoidal shaped, Gram positive, nonmotile, and asporogenous (Sahin et al., 2021). They are obligately heterofermentative, and metabolize glucose to produce lactic acid, ethanol, and CO_2_. They have pentose and pentitol metabolism pathway, which metabolizes L‐/D‐arabinose, D‐ribose, and D‐xylose to produce acetate and lactate. They also metabolize citrate and malate to produce diacetyl, acetoin, and 2,3‐butanediol, which is an important reaction to produce aroma in dairy products, wine, and other foods (Cogan & Jordan, 1994). They are observed to be dominant in the early and middle stages of kimchi fermentation, and they are selected as starter cultures owing to their good acid‐producing activity and contribution to flavor (Jung et al., 2012). The strains of *Leuconostoc* do not have a strong tolerance to acid and salt, perhaps it is the reason that in the later stage of pickling with high salt concentration, the dominant genus became *Lactobacillus*. The *Debaryomyces* are reported to be dominant in fermented dairy and meat products, they can grow under high salt, low water activities (Aw), and other adverse conditions, with remarkable abilities to produce endo‐ and exopeptidases to degrade proteins into free amino acids, as well as lipases to hydrolyze fat into fatty acids, and thus contribute to flavors (Breuer & Harms, 2006; Flores & Toldra, 2011). The *Kazachstania* is also normally found in high‐salt foods, such as kimchi, Chinese bean peppers, and fermented fish, they are inferred to be related to aroma components, however, its precise roles remain to be investigated (Punyauppa‐path et al., 2022).

This study determined that glucose and fructose were the two major monosaccharides in tuber samples during the pickling. Undoubtedly, glucose is the optimum sugar for most microorganisms. It is worth noting that the concentration of fructose in raw tuber and its succession process in pickling stages are close to that of glucose, which suggests that the metabolism of fructose by microorganisms probably also accounts for a large proportion of sugar metabolism. Microorganisms can metabolize fructose in the following ways: (1) fructose is catalyzed by hexokinase to produce fructose‐6‐phosphate, which is then degraded through glycolysis. (2) Some microorganisms, typically the *Leuconostoc* cells, have mannitol dehydrogenase, which can directly catalyze fructose to mannitol (Sahin et al., 2021). (3) Some lactic acid bacteria (LAB) have fructophilic characteristics, that is, fructose is their optimum substrate and preferentially metabolized than glucose. Generally speaking, fructophilic LAB are heterofermentative strains because their glucose and fructose metabolism have three pathways leading to the end product of lactic acid, acetic acid, and ethanol, respectively, while homofermentative cells do not have the pathway to ethanol. The fructophilic LAB lack alcohol dehydrogenase (ADH) and/or acetaldehyde dehydrogenase (ALDH) or their activities are relatively lower than that of heterofermentative LAB, which is the important difference between fructophilic and heterofermentative LAB. Therefore, the metabolic pathway of producing ethanol from Acetyl‐1‐P in fructophilic LAB is blocked and converted to the pathway of producing acetic acid with the catalysis of acetate kinase, simultaneously, the fructose, pyruvate, and O_2_ act as the electron acceptor to maintain the balance of NAD/NADH. It is reported that more than 69% of fructophilic LAB are related to *Leuconostoc* spp (Endo et al., 2018).

This study reveals the main VFCs in salt‐reducing pickling *zhacai* include isothiocyanates, aldehydes, and dimethyl trisulfide. The VFCs in traditional pickling *zhacai* have been investigated, and Lin and Hua (1986) reported that the characteristic aroma is formed by components such as isothiocyanates, nitriles, and dimethyl trisulfide; esters, heterocyclic compounds, and other oxygen‐containing compounds also contribute to aroma, thus forming a unique pungent aroma. Liu (2009) reported that the main volatile components in *zhacai* pickling are allyl isothiocyanate, benzyl isothiocyanate, dimethyl trisulfide, and phenylpropionitrile. On the whole, our results on salt‐reducing *zhacai* are consistent with those of traditional *zhacai*. The isothiocyanate is generated from the degradation reaction of glucosinolates, a kind of characteristic components present in mustard tuber (*Cruciferae* family plant). Glucosinolates and endogenous myrosinase are located in different locations within the tuber cells, and extrusion or pressing can break cells, facilitate contact, and enable hydrolysis of the glucosinolates (Tripathi & Mishra, 2007). The β‐D‐thioglucose is removed from glucosinolates, leading to the formation of thiohydroximate‐O‐sulphonate (an organic aglucone), aglucones are unstable and undergo Lossen rearrangement to yield isothiocyanates (ITCs) at neutral pH, while acidic pH and Fe^2+^ favor the production of nitriles (Tripathi & Mishra, 2007). In addition to endogenous myrosinase, degradation of glucosinates has been reported via many microorganisms; these include *Lactobacillus plantarum* KW30, *Lactobacillus agilis* R16, and *Pediococcus pentosaceus* UM116P (Tian et al., 2018), and the fungi *Aspergillus clavatus* and *Fusarium oxysporum* (Smits et al., 1993). This study identified eight ITCs in *zhacai*, with allyl isothiocyanate (E1) expressed as the dominant component, which was consistent with previous studies (Liu, 2009). Allyl isothiocyanate elicits strong pungent smells, and also has anti‐inflammatory, anticancer, antibacterial, and other biological activities (Traka & Mithen, 2009), affording *zhacai* a variety of physiological and health benefits. Two nitriles are detected, 3‐butenenitrile (N1) and benzenepropanenitrile (N2), which are probably also the degrading products of glucosinolates owing to acidic brine conditions, whereas consuming a large amount of nitriles probably exert negative effects on health, such as affecting thyroid function, hepatotoxicity, and nephrotoxicity (Tripathi & Mishra, 2007). It is necessary to develop methods for regulating the glucosinolates' degrading products to be mainly isothiocyanates and avoiding the formation of nitriles for improving the health benefit of *zhacai*. The second major category of *zhacai* VFCs was the aldehydes. There are two possible pathways via which aldehydes are produced. Firstly, there is the oxidation of fatty acids. For example, octanoic acid (C1) and linolenic acid (C2) were detected in the raw tubers, and their oxidation reaction probably produce hexanal (A1) and (E)‐2‐heptenal (A3). Secondly, there is the metabolic activity of microorganisms. PICRUSt2 function prediction (Figure 7) highlighted synthetic pathways for palmitate, oleate, palmitoleate, (5Z)‐dodec‐5‐enoate, and stearate, as well as metabolic pathways for fatty acid elongation, β‐oxidation, and salvage in the bacterial community. Palmitate and stearate synthetic pathways and β‐oxidation pathways were also apparent in the fungal community. On the other hand, the Ehrlich pathway in yeast is reported to be an important way to produce aldehydes—amino acids are converted to α‐keto acids by transamination, then decarboxylated to produce aldehydes, and reduced to alcohols (Hazelwood et al., 2008). Therefore, it can be inferred that bacteria and fungi probably play a role in producing aldehydes (and even subsequently alcohols).

**FIGURE 8:**
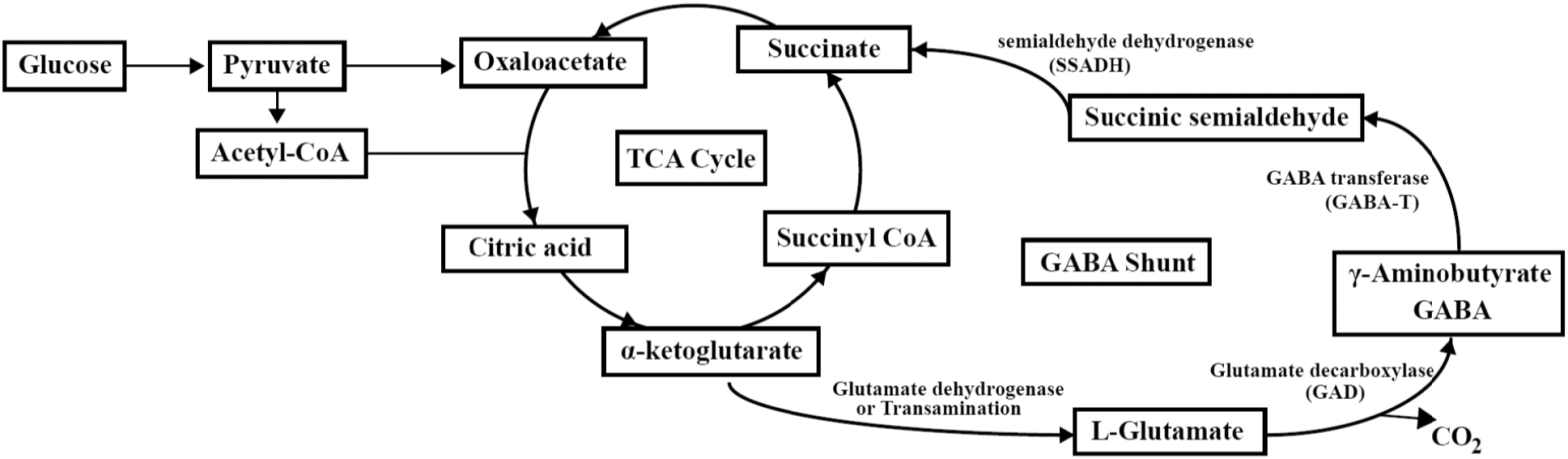
Major metabolic pathway of GABA (GABA shunt pathway).

This study observed that the concentration of γ‐aminobutyric acid (GABA) in tuber samples increased significantly during the pickling process. Microorganisms can produce GABA through the GABA shunt pathway (Figure 8), the alpha‐ketoglutarate generated by the TCA cycle is converted to glutamate, and the glutamate is catalyzed by glutamate decarboxylase (GAD) to produce GABA, which is subjected to further reaction to produce succinic semialdehyde and succinate, and then succinate enters the TCA cycle (Sarasa et al., 2020). LAB, such as *Lactobacillus plantarum* and *Lactobacillus brevis* and the yeast *Saccharomyces cerevisiae* isolated from Chinese *paocai* are identified to produce GABA (Li et al., 2008; Zhang et al., 2017, 2020). The reaction of producing GABA is related to microbial physiological mechanism to tolerate acid environment. When in an acidic environment, there is more H^+^ inside microbial cells, the glutamate in the environment is transported into cells and subjected to decarboxylation reaction under the catalysis of GAD enzyme with the consumption of intracellular H^+^, the product GABA is excluded from the cell through antiporter, and it is an alkaline compound that neutralizes acids in the environment (Sarasa et al., 2020). Therefore, this reaction can help microbial cells balance the acid inside and outside the cells and overcome the acidic environment in fermented food. GABA is a major inhibitory neurotransmitter in the mammal central nervous system and has physiological effects including antihypertensive, antiepileptic, antidepressant, and antidiabetic effects (Zhang et al., 2017), thus affording *zhacai* a variety of physiological and health benefits.

## 5 CONCLUSION

The succession patterns of microbial communities during the salt‐reducing pickling process of *zhacai* were elucidated using the PacBio Sequel platform. In the bacterial community, the average number of nonsingletons per sample was 4989.47 ± 1087.24 and the sequencing length was 1400 bp. Eleven phyla and 148 genera were identified. In the fungal community, the average number of nonsingletons per sample was 5811.65 ± 2104.19 and the sequencing length was 1200 bp. Two phyla and 60 genera were identified. During the four stages of pickling, the dominant bacterial genera were *Leuconostoc*, *Lactobacillus*, *Leuconostoc*, and *Lactobacillus*, while the dominant fungal genera were *Aspergillus*, *Kazachstania*, *Debaryomyces*, and *Debaryomyces*. The simultaneous development of flavor components in *zhacai* was also demonstrated. There were 32 main flavor components (5 organic acids, 19 VFCs, 3 monosaccharides, and 5 amino acids). Correlation heat mapping and O2PLS analysis showed that the flora playing key roles in the formation of flavor components included 14 genera of bacteria (*Leuconostoc*, *Clostridium*, *Devosia*, *Lactococcus*, *Pectobacterium*, *Sphingobacterium*, *Serratia*, *Stenotrophomonas*, *Halanaerobium*, *Tetragenococcus*, *Chromohalobacter*, *Klebsiella*, *Acidovorax*, and *Acinetobacter*), and 3 genera of fungi (*Filobasidium*, *Malassezia*, and *Aspergillus*). The bacteria and fungi probably produce *zhacai* flavor components through the metabolic activity of saccharides, glucosinolates, fatty acids, and amino acids.

## CONFLICT OF INTEREST STATEMENT

The authors declare that they have no known competing financial interests or personal relationships that could have appeared to influence the work reported in this paper.

## ETHICS STATEMENT

This study does not involve any human or animal testing.

## Supporting information


Appendix S1.

## Data Availability

All relevant data are within the manuscript and its Supporting Information files. All sequence reads have been deposited at the NCBI Sequence Read Archive. (SRA) under project accession numbers PRJNA725670 and PRJNA725681. Please see https://www.ncbi.nlm.nih.gov/bioproject/PRJNA725670 and https://www.ncbi.nlm.nih.gov/bioproject/PRJNA725681.

## References

[fsn33297-bib-0001] Abarenkov, K. , Henrik Nilsson, R. , Larsson, K. H. , Alexander, I. J. , Eberhardt, U. , Erland, S. , Høiland, K. , Kjøller, R. , Larsson, E. , Pennanen, T. , Sen, R. , Taylor, A. F. , Tedersoo, L. , Ursing, B. M. , Vrålstad, T. , Liimatainen, K. , Peintner, U. , & Kõljalg, U. (2010). The UNITE database for molecular identification of fungi—Recent updates and future perspectives. New Phytologist, 186, 281–285. 10.1111/j.1469-8137.2009.03160.x 20409185

[fsn33297-bib-0002] Bokulich, N. A. , Kaehler, B. D. , Rideout, J. R. , Dillon, M. , Bolyen, E. , Knight, R. , Huttley, G. A. , & Gregory Caporaso, J. (2018). Optimizing taxonomic classification of marker‐gene amplicon sequences with QIIME 2's q2‐feature‐classifier plugin. Microbiome, 1, 90. 10.7287/peerj.preprints.3208v1 PMC5956843 29773078

[fsn33297-bib-0003] Bolyen, E. , Rideout, J. R. , Dillon, M. R. , Bokulich, N. A. , Abnet, C. C. , al‐Ghalith, G. A. , Alexander, H. , Alm, E. J. , Arumugam, M. , Asnicar, F. , Bai, Y. , Bisanz, J. E. , Bittinger, K. , Brejnrod, A. , Brislawn, C. J. , Brown, C. T. , Callahan, B. J. , Caraballo‐Rodríguez, A. M. , Chase, J. , … Caporaso, J. G. (2019). Reproducible, interactive, scalable and extensible microbiome data science using QIIME 2. Nature Biotechnology, 37, 852–857. 10.1038/s41587-019-0209-9 PMC7015180 31341288

[fsn33297-bib-0004] Breuer, U. , & Harms, H. (2006). *Debaryomyces hansenii*—An extremophilic yeast with biotechnological potential. Yeast, 23(6), 415–437. 10.1002/yea.1374 16652409

[fsn33297-bib-0005] Cogan, T. M. , & Jordan, K. N. (1994). Metabolism of *Leuconostoc* bacteria. Journal of Dairy Science, 77, 2704–2717. 10.3168/jds.S0022-0302(94)77213-1

[fsn33297-bib-0007] Douglas, G. M. , Maffei, V. J. , Zaneveld, J. R. , Yurgel, S. N. , Brown, J. R. , Taylor, C. M. , Huttenhower, C. , & Langille, M. G. I. (2020). PICRUSt2 for prediction of metagenome functions. Nature Biotechnology, 38, 685–688. 10.1038/s41587-020-0548-6 PMC7365738 32483366

[fsn33297-bib-0008] Endo, A. , Maeno, S. , Tanizawa, Y. , Kneifel, W. , Arita, M. , Dicks, L. , & Salminen, S. (2018). Fructophilic lactic acid bacteria, a unique group of fructose‐fermenting microbes. Applied and Environmental Microbiology, 84, e01290‐18. 10.1128/AEM.01290-18 30054367 PMC6146980

[fsn33297-bib-0009] Fan, Y. H. , Shen, J. J. , & Dong, D. W. (2016). Development status and research prospect of mustard vegetables industry. Journal of Agriculture, 6, 65–71.

[fsn33297-bib-0010] Fichot, E. B. , & Norman, R. S. (2013). Microbial phylogenetic profiling with the Pacific biosciences sequencing platform. Microbiome, 1, 10. 10.1186/2049-2618-1-10 24450498 PMC3971627

[fsn33297-bib-0011] Flores, M. , & Toldra, F. (2011). Microbial enzymatic activities for improved fermented meats. Trends in Food Science and Technology, 22(2–3), 81–90. 10.1016/j.tifs.2010.09.007

[fsn33297-bib-0012] Hazelwood, L. A. , Daran, J. M. , Maris, A. V. , & Pronk, J. T. (2008). The Ehrlich pathway for fusel alcohol production: A century of research on *Saccharomyces cerevisiae* metabolism. Applied and Environmental Microbiology, 8, 2259–2266. 10.1128/aem.02625-07 PMC2293160 18281432

[fsn33297-bib-0013] He, Y. C. , & Hou, Y. (2012). The research of *Zhacai* food industrialization. Food and Fermentation Technology, 48, 19–22.

[fsn33297-bib-0014] He, Y. C. , Zhou, B. Q. , & Liu, D. J. (2013). The overview of traditional Fuling *Zhacai*'s processes. Food and Fermentation Technology, 49, 57–60.

[fsn33297-bib-0015] Huang, Z. R. , Hong, J. L. , Xu, J. X. , Li, L. , Guo, W. L. , Pan, Y. Y. , Chen, S. J. , Bai, W. D. , Rao, P. F. , Ni, L. , Zhao, L. N. , Liu, B. , & Lv, X. C. (2018). Exploring core functional microbiota responsible for the production of volatile flavour during the traditional brewing of *Wuyi* Hong Qu glutinous rice wine. Food Microbiology, 76, 487–496. 10.1016/j.fm.2018.07.014 30166178

[fsn33297-bib-0016] Jung, J. Y. , Lee, S. H. , Lee, H. J. , Seo, H. Y. , Park, W. S. , & Jeon, C. O. (2012). Effects of *Leuconostoc mesenteroides* starter cultures on microbial communities and metabolites during kimchi fermentation. International Journal of Food Microbiology, 153, 378–387. 10.1016/j.ijfoodmicro.2011.11.030 22189023

[fsn33297-bib-0017] Kawai, M. , Okiyama, A. , & Ueda, Y. (2002). Taste enhancements between various amino acids and IMP. Chemical Senses, 8, 739–745. 10.1093/chemse/27.8.739 12379598

[fsn33297-bib-0018] Li, H. X. , Gao, D. D. , Cao, Y. S. , & Xu, H. Y. (2008). A high γ‐aminobutyric acid‐producing *Lactobacillus brevis* isolated from Chinese traditional *paocai* . Annals of Microbiology, 58, 649–653. 10.1007/BF03175570

[fsn33297-bib-0019] Li, Q. , Li, Y. D. , Luo, Y. , Zhang, Y. Y. , Chen, Y. , Lin, H. Y. , Wang, K. , Huang, J. , & Liu, Z. (2019). Shifts in diversity and function of the bacterial community during the manufacture of Fu brick tea. Food Microbiology, 80, 70–76. 10.1016/j.fm.2019.01.001 30704598

[fsn33297-bib-0020] Li, X. G. (2003). Discussion on the main composition change in the salting process of pickle. China Brewing, 3, 9–12.

[fsn33297-bib-0021] Lin, Z. K. , & Hua, Y. F. (1986). A study on the volatile flavor constituents of Sichuan preserved vegetable (*Brassica juncea* czern. Et coss.). Acta Botanica Sinica, 3, 299–306.

[fsn33297-bib-0022] Lioe, H. N. , Apriyantono, A. , Takara, K. , Wada, K. , Naoki, H. , & Yasuda, M. (2004). Low molecular weight compounds responsible for savory taste of Indonesian soy sauce. Journal of Agricultural and Food Chemistry, 19, 5950–5956. 10.1021/jf049230d 15366848

[fsn33297-bib-0023] Liu, M. C. (2009). Study on the formation and changes of volatile compounds in pickled mustard tuber during the pickling process, M.a. Thesis. Chongqing University.

[fsn33297-bib-0024] Martin, M. (2011). Cutadapt removes adapter sequences from high‐throughput sequencing reads. EMBnet Journal, 17, 10–12. 10.14806/ej.17.1.200

[fsn33297-bib-0025] Ministry of Agriculture and Rural Affairs of the People's Republic of China . (2012). Agricultural Standard‐Green food Pickled vegetables NY/T 437–2012 . Retrieved from http://down.foodmate.net/standard/sort/5/36195.html

[fsn33297-bib-0026] Punyauppa‐path, S. , Kiatprasert, P. , Punyauppa‐path, P. , Rattanachaikunsopon, P. , Khunnamwong, P. , Limtong, S. , & Srisuk, N. (2022). Distribution of *Kazachstania* yeast in Thai traditional fermented fish (Plaa‐Som) in northeastern Thailand. Journal of Fungi, 8, 1029. 10.3390/jof8101029 36294595 PMC9605060

[fsn33297-bib-0027] Roberts, R. J. , Carneiro, M. O. , & Schatz, M. C. (2013). The advantages of SMRT sequencing. Genome Biology, 7, 405. 10.1186/gb-2013-14-6-405 PMC3953343 23822731

[fsn33297-bib-0028] Rognes, T. , Flouri, T. , Nichols, B. , Quince, C. , & Mahé, F. (2016). VSEARCH: A versatile open source tool for metagenomics. PeerJ, 4, e2584. 10.7717/peerj.2584 27781170 PMC5075697

[fsn33297-bib-0029] Sahin, A. W. , Rice, T. , & Coffey, A. (2021). Genomic analysis of *Leuconostoc citreum* TR116 with metabolic reconstruction and the effects of fructose on gene expression for mannitol production. International Journal of Food Microbiology, 354, 109327. 10.1016/j.ijfoodmicro.2021.109327 34247022

[fsn33297-bib-0030] Sarasa, S. B. , Mahendran, R. , Muthusamy, G. , Thankappan, B. , Selta, D. R. F. , & Angayarkanni, J. (2020). A brief review on the non‐protein amino acid, gamma‐amino butyric acid (GABA): Its production and role in microbes. Current Microbiology, 77, 534–544. 10.1007/s00284-019-01839-w 31844936

[fsn33297-bib-0031] Segata, N. , Izard, J. , Waldron, L. , Gevers, D. , Miropolsky, L. , Garrett, W. S. , & Huttenhower, C. (2011). Metagenomic biomarker discovery and explanation. Genome Biology, 12, R60. 10.1186/gb-2011-12-6-r60 21702898 PMC3218848

[fsn33297-bib-0032] Smits, J. P. , Knol, W. , & Bol, J. (1993). Glucosinolate degradation by *aspergillus clavatus* and *Fusarium oxysporum*, in liquid and solid‐state fermentation. Applied Microbiology and Biotechnology, 5, 696–701. 10.1007/BF00182812

[fsn33297-bib-0033] Stoddard, S. F. , Smith, B. J. , Hein, R. , Roller, B. R. K. , & Schmidt, T. M. (2015). *rrn*DB: Improved tools for interpreting rRNA gene abundance in bacteria and archaea and a new foundation for future development. Nucleic Acids Research, D1, D593–D598. 10.1093/nar/gku1201 PMC4383981 25414355

[fsn33297-bib-0034] Tedersoo, L. , Anslan, S. , Bahram, M. , Kõljalg, U. , & Abarenkov, K. (2020). Identifying the 'unidentified' fungi: A global‐scale long‐read third‐generation sequencing approach. Fungal Diversity, 1, 273–293. 10.1007/s13225-020-00456-4

[fsn33297-bib-0035] Tian, S. C. , Liu, X. D. , Lei, P. , Zhang, X. H. , & Shan, Y. J. (2018). Microbiota: A mediator to transform glucosinolate precursors in cruciferous vegetables to the active isothiocyanates. Journal of the Science of Food and Agriculture, 4, 1255–1260. 10.1002/jsfa.8654 28869285

[fsn33297-bib-0036] Traka, M. , & Mithen, R. (2009). Glucosinolates, isothiocyanates and human health. Phytochemistry Reviews, 8, 269–282. 10.1007/s11101-008-9103-7

[fsn33297-bib-0037] Tripathi, M. K. , & Mishra, A. S. (2007). Glucosinolates in animal nutrition: A review. Animal Feed Science and Technology, 132, 1–27. 10.1016/j.anifeedsci.2006.03.003

[fsn33297-bib-0038] Van Gemert, L. J. , & Nettenbreijer, A. H. (2011). Compilations of odour threshold values in air, water and other media (second enlarged and revised edition). Oliemans Punter & Partners BV.

[fsn33297-bib-0039] Wakinaka, T. , Iwata, S. , Takeishi, Y. , Watanabe, J. , Mogi, Y. , Tsukioka, Y. , & Shibata, Y. (2019). Isolation of halophilic lactic acid bacteria possessing aspartate decarboxylase and application to fish sauce fermentation starter. International Journal of Food Microbiology, 292, 137–143. 10.1016/j.ijfoodmicro.2018.12.013 30599453

[fsn33297-bib-0040] Wang, Z. M. (2016). Correlation between structural shifts of microbiota and compositions of flavors during the stage of acetic acid fermentation of Zhenjiang aromatic vinegar, Ph.D. Thesis. Jiangnan University.

[fsn33297-bib-0041] Yang, J. X. , Li, F. Z. , Zhang, Y. L. , & He, Z. F. (2021). Metagenomic analysis of microbial community succession during the pickling process of *Zhacai* (preserved mustard tuber) and its correlation with *Zhacai* biochemical indices. Journal of the Science of Food and Agriculture, 4, 1646–1658. 10.1002/jsfa.10785 32888329

[fsn33297-bib-0042] Yuan, F. (2008). Analysis on volatility composition and sensory of tuber mustard and pickled tuber mustard, M.a. Thesis. Southwest University.

[fsn33297-bib-0043] Zhang, Q. , Sun, Q. , Tan, X. , Zhang, S. M. , Zeng, L. , Tang, J. , & Xiang, W. L. (2020). Characterization of γ‐aminobutyric acid (GABA)‐producing *Saccharomyces cerevisiae* and coculture with *Lactobacillus plantarum* for mulberry beverage brewing. Journal of Bioscience and Bioengineering, 129, 447–453. 10.1016/j.jbiosc.2019.10.001 31678068

[fsn33297-bib-0044] Zhang, Q. , Zeng, L. , Tan, X. , Tang, J. , & Xiang, W. L. (2017). An efficient γ‐aminobutyric acid (GABA) producing and nitrite reducing ability of *Lactobacillus plantarum* BC114 isolated from Chinese *paocai* . Food Science and Technology Research, 23, 749–755. 10.3136/fstr.23.749

[fsn33297-bib-0045] Zhao, C. , Su, W. , Mu, Y. C. , Jiang, L. , & Mu, Y. (2020). Correlations between microbiota with physicochemical properties and volatile flavor components in black glutinous rice wine fermentation. Food Research International, 138, 109800. 10.1016/j.foodres.2020.109800 33288182

[fsn33297-bib-0046] Zheng, X. J. , Liu, F. , Shi, X. W. , Wang, B. , Li, K. X. , Li, B. K. , & Zhuge, B. (2018). Dynamic correlations between microbiota succession and flavor development involved in the ripening of Kazak artisanal cheese. Food Research International, 105, 733–742. 10.1016/j.foodres.2017.12.007 29433268

[fsn33297-bib-0047] Zhuang, K. J. , Wu, N. , Wang, X. C. , Wu, X. G. , Wang, S. , Long, X. W. , & Wei, X. (2016). Effects of 3 feeding modes on the volatile and nonvolatile compounds in the edible tissues of female Chinese mitten crab (*Eriocheir sinensis*). Journal of Food Science, 81, 968–981. 10.1111/1750-3841.13229 26919186

