# Supplementary material for: Analysis of the microbial community structure and flavor components succession during salt‐reducing pickling process of zhacai (preserved mustard tuber)
Source: Food Sci Nutr. 2023 Apr 17;11(6):3154–70. doi: 10.1002/fsn3.3297 (PMC10261794; doi:10.1002/fsn3.3297)
Supplement: Supplementary file 1 — Appendix S1. [file FSN3-11-3154-s001.zip › ═╝║═▒φ/Figure 2. The variation process of zhacai VFCs concentration.docx]

Figure 2. The variation process of *zhacai* VFCs concentration and OAV during pickling process

E1: Allyl isothiocyanate; E2: 2-Phenylethyl isothiocyanate; E3: 3-Butenyl isothiocyanate; E5: Butyl isothiocyanate; E10: Octanoic acid ethyl ester; A1: Hexanal; A4: (E,E)-2,4-Heptadienal; A5: Phenylacetaldehyde; A6: (E)-2-Octenal; A7: Nonanal; A8: (E,E)-2,4-Decadienal; A9: (E,Z)- 2,6-Nonadienal; A11: 2-Undecenal; L1: 3-Methyl-1-butanol; L2: (E)-2-Octen-1-ol; K1: 3,5-Octadien-2-one; C2: Linolenic acid; N2: Benzenepropanenitrile; O4: Dimethyl trisulfide.

| **Esters** |  | A9 | (E,Z)- 2,6-Nonadienal |
| --- | --- | --- | --- |
| E1 | Allyl isothiocyanate | A10 | 4-Ethylbenzaldehyde |
| E2 | 2-Phenylethyl isothiocyanate | A11 | 2-Undecenal |
| E3 | 3-Butenyl isothiocyanate | **Alcohols** |  |
| E4 | 3-(Methylthio)propyl isothiocyanate | L1 | 3-Methyl-1-butanol |
| E5 | Butyl isothiocyanate | L2 | (E)-2-Octen-1-ol |
| E6 | Isobutyl isothiocyanate | L3 | 1-Octanol |
| E7 | 3-Methylbutyl isothiocyanate | L4 | Phenylethyl alcohol |
| E8 | Pentyl isothiocyanate | **Ketones** |  |
| E9 | Pentadecanoic acid ethyl ester | K1 | 3,5-Octadien-2-one |
| E10 | Octanoic acid ethyl ester | K2 | 6,10-Dimethyl-5,9-undecadien-2-one |
| E11 | Tridecanoic acid methyl ester | **Acids** |  |
| E12 | (Z)-9-Octadecenoic acid methyl ester | C1 | Octanoic acid |
| E13 | 9,12-Octadecadienoic acid(Z,Z)-methyl ester | C2 | Linolenic acid |
| **Aldehyde** |  | **Nitriles** |  |
| A1 | Hexanal | N1 | 3-Butenenitrile |
| A2 | (E)- 2-Hexenal | N2 | Benzenepropanenitrile |
| A3 | (E)-2-Heptenal | **Others** |  |
| A4 | (E,E)-2,4-Heptadienal | O1 | 2-Ethylfuran |
| A5 | Phenylacetaldehyde | O2 | 2-Pentylfuran |
| A6 | (E)-2-Octenal | O3 | (Z)-2-(2-Pentenyl) furan |
| A7 | Nonanal | O4 | Dimethyl trisulfide |
| A8 | (E,E)-2,4-Decadienal |  |  |
