# Supplementary material for: Analysis of the microbial community structure and flavor components succession during salt‐reducing pickling process of zhacai (preserved mustard tuber)
Source: Food Sci Nutr. 2023 Apr 17;11(6):3154–70. doi: 10.1002/fsn3.3297 (PMC10261794; doi:10.1002/fsn3.3297)
Supplement: Supplementary file 1 — Appendix S1. [file FSN3-11-3154-s001.zip › ═╝║═▒φ/Figure 7. The KEGG pathway involved with the fatty acid metabolism predicted by PICRUSt2.docx]

Figure 7. The KEGG pathway involved with the fatty acid metabolism predicted by PICRUSt2 in bacterial (A) and fungal (B) community

For (A) Bacteria:

FASYN-ELONG-PWY: fatty acid elongation - saturated

PWY-5971: palmitate biosynthesis II [bacteria and plants]

PWY-7664: oleate biosynthesis IV [anaerobic]

PWY-6282: palmitoleate biosynthesis I [from (5Z)-dodec-5-enoate]

PWY0-862: [5Z]-dodec-5-enoate biosynthesis

PWY-5989: stearate biosynthesis II [bacteria and plants]

FASYN-INITIAL-PWY: superpathway of fatty acid biosynthesis initiation [*E. coli*]

FAO-PWY: fatty acid & β-oxidation I

PWY-7094: fatty acid salvage

For (B) Fungi:

PWY-7288: fatty acid & β-oxidation [peroxisome, yeast]

PWY-6837: fatty acid β-oxidation V [unsaturated, odd number, di-isomerase-dependent]

PWY-5994: palmitate biosynthesis I [animals and fungi]

PWY30-355: stearate biosynthesis III [fungi]
