# Supplementary material for: Analysis of the microbial community structure and flavor components succession during salt‐reducing pickling process of zhacai (preserved mustard tuber)
Source: Food Sci Nutr. 2023 Apr 17;11(6):3154–70. doi: 10.1002/fsn3.3297 (PMC10261794; doi:10.1002/fsn3.3297)
Supplement: Supplementary file 1 — Appendix S1. [file FSN3-11-3154-s001.zip › ═╝║═▒φ/S1 Table. Soluble monosaccharide concentrations during the zhacai pickling process.docx]

S1 Table. Monosaccharide concentrations during the *zhacai* pickling process

| Monosaccharide | Concentration of monosaccharide (μg·kg ^-1^) | | | | | | | | | | | | | | | | |
| --- | --- | --- | --- | --- | --- | --- | --- | --- | --- | --- | --- | --- | --- | --- | --- | --- | --- |
|  | S0 | S11 | S12 | S21 | S22 | S23 | S24 | S31 | S32 | S33 | S34 | S41 | S42 | S43 | S44 | S45 | S46 |
| Fucose (Fuc) | 0.044  ±0.008 | 0.072  ±0.009 | 0.077  ±0.012 | 0.049  ±0.016 | 0.046  ±0.014 | 0.064  ±0.011 | 0.073  ±0.012 | 0.085  ±0.017 | 0.087  ±0.012 | 0.094  ±0.022 | 0.112  ±0.015 | 0.126  ±0.010 | 0.143  ±0.007 | 0.157  ±0.008 | 0.164  ±0.009 | 0.196  ±0.021 | 0.197  ±0.017 |
| Arabinose (Ara) | 0.005  ±0.002 | 0.006  ±0.001 | 0.006  ±0.001 | 0.007  ±0.001 | 0.011  ±0.002 | 0.029  ±0.003 | 0.033  ±0.005 | 0.054  ±0.011 | 0.072  ±0.006 | 0.076  ±0.009 | 0.087  ±0.007 | 0.118  ±0.026 | 0.154  ±0.011 | 0.230  ±0.017 | 0.302  ±0.015 | 0.350  ±0.009 | 0.346  ±0.016 |
| Galactose (Gal) | 0.023  ±0.008 | 0.045  ±0.012 | 0.056  ±0.010 | 0.135  ±0.017 | 0.377  ±0.053 | 0.615  ±0.028 | 0.812  ±0.043 | 1.066  ±0.118 | 1.288  ±0.150 | 1.447  ±0.124 | 1.761  ±0.132 | 1.935  ±0.158 | 2.189  ±0.143 | 2.235  ±0.085 | 2.485  ±0.147 | 2.464  ±0.190 | 2.332  ±0.185 |
| Glucose (Glc) | 9.328  ±0.361 | 8.118  ±0.234 | 8.623  ±0.457 | 7.132  ±0.173 | 6.919  ±0.636 | 5.894  ±0.564 | 5.173  ±0.230 | 5.592  ±0.323 | 5.357  ±0.519 | 4.784  ±0.600 | 4.245  ±0.454 | 4.032  ±0.194 | 4.375  ±0.528 | 3.227  ±0.191 | 3.121  ±0.130 | 3.076  ±0.179 | 3.362  ±0.202 |
| Xylose (Xyl) | - ^a^ | - | - | - | 0.053  ±0.011 | 0.302  ±0.053 | 0.387  ±0.058 | 0.343  ±0.044 | 0.410  ±0.025 | 0.402  ±0.032 | 0.549  ±0.081 | 0.560  ±0.051 | 0.542  ±0.058 | 0.619  ±0.033 | 0.636  ±0.067 | 0.617  ±0.022 | 0.655  ±0.046 |
| Mannose (Man) | - | - | - | - | 0.034  ±0.008 | 0.041  ±0.010 | 0.073  ±0.009 | 0.084  ±0.008 | 0.100  ±0.012 | 0.116  ±0.010 | 0.119  ±0.018 | 0.127  ±0.011 | 0.139  ±0.015 | 0.135  ±0.018 | 0.155  ±0.009 | 0.164  ±0.012 | 0.155  ±0.033 |
| Fructose (Fru) | 9.263  ±0.409 | 8.447  ±0.535 | 7.567  ±0.556 | 7.337  ±0.643 | 7.163  ±0.422 | 7.343  ±0.472 | 7.403  ±0.635 | 6.890  ±0.671 | 6.387  ±0.572 | 5.793  ±0.735 | 5.877  ±0.858 | 5.423  ±0.641 | 5.277  ±0.420 | 5.670  ±0.328 | 4.923  ±0.662 | 4.103  ±0.431 | 4.350  ±0.375 |
| Ribose (Rib) | 0.122  ±0.014 | - | - | - | - | - | 0.173  ±0.043 | 0.155  ±0.032 | 0.085  ±0.012 | 0.064  ±0.009 | 0.043  ±0.010 | 0.035  ±0.008 | 0.024  ±0.007 | 0.021  ±0.007 | 0.017  ±0.006 | 0.012  ±0.003 | 0.010  ±0.002 |
| Galacturonic Acid (Gal-UA) | 0.089  ±0.013 | 0.084  ±0.010 | 0.073  ±0.009 | 0.084  ±0.010 | 0.077  ±0.010 | 0.075  ±0.007 | 0.064  ±0.009 | 0.083  ±0.010 | 0.075  ±0.012 | 0.058  ±0.006 | 0.051  ±0.005 | 0.074  ±0.012 | 0.067  ±0.012 | 0.052  ±0.009 | 0.051  ±0.006 | 0.067  ±0.009 | 0.070  ±0.005 |
| Guluronic Acid (Gul-UA) | 0.044  ±0.003 | 0.041  ±0.005 | 0.045  ±0.003 | 0.042  ±0.002 | 0.036  ±0.004 | 0.034  ±0.003 | 0.029  ±0.005 | 0.031  ±0.006 | 0.027  ±0.005 | 0.024  ±0.004 | 0.022  ±0.005 | 0.020  ±0.004 | 0.018  ±0.005 | 0.019  ±0.005 | 0.019  ±0.006 | 0.018  ±0.002 | 0.019  ±0.003 |
| Glucuronic Acid (Glc-UA) | 0.054  ±0.010 | 0.071  ±0.012 | 0.056  ±0.007 | 0.039  ±0.007 | 0.036  ±0.010 | 0.029  ±0.004 | 0.026  ±0.005 | 0.028  ±0.006 | 0.029  ±0.007 | 0.024  ±0.004 | 0.022  ±0.005 | 0.021  ±0.003 | 0.025  ±0.005 | 0.026  ±0.004 | 0.028  ±0.009 | 0.027  ±0.004 | 0.029  ±0.006 |

a: "-", Undetected
