# Supplementary material for: Analysis of the microbial community structure and flavor components succession during salt‐reducing pickling process of zhacai (preserved mustard tuber)
Source: Food Sci Nutr. 2023 Apr 17;11(6):3154–70. doi: 10.1002/fsn3.3297 (PMC10261794; doi:10.1002/fsn3.3297)
Supplement: Supplementary file 1 — Appendix S1. [file FSN3-11-3154-s001.zip › ═╝║═▒φ/S2 Figure. Diversity indices of (A) bacteria and (B) fungi.docx]

**S2 Figure. Diversity indices of (A) bacteria and (B) fungi, and (C) principal component analysis (PCA) plots of both communities**
