# Supplementary material for: Analysis of the microbial community structure and flavor components succession during salt‐reducing pickling process of zhacai (preserved mustard tuber)
Source: Food Sci Nutr. 2023 Apr 17;11(6):3154–70. doi: 10.1002/fsn3.3297 (PMC10261794; doi:10.1002/fsn3.3297)
Supplement: Supplementary file 1 — Appendix S1. [file FSN3-11-3154-s001.zip › ═╝║═▒φ/S2 Table. Volatile flavor component concentrations during the zhacai pickling process.docx]

**S2 Table. Volatile flavor component concentrations during the *zhacai* pickling process**

| Number | Volatile compounds | CAS Number | Retentation  indices | Concentration of VFCs（μg·kg ^-1^） | | | | | | | | | | | | | | | | |
| --- | --- | --- | --- | --- | --- | --- | --- | --- | --- | --- | --- | --- | --- | --- | --- | --- | --- | --- | --- | --- |
|  |  |  |  | S0 | S11 | S12 | S21 | S22 | S23 | S24 | S31 | S32 | S33 | S34 | S41 | S42 | S43 | S44 | S45 | S46 |
| **Esters** | |  |  |  |  |  |  |  |  |  |  |  |  |  |  |  |  |  |  |  |
| E1 | Allyl isothiocyanate | 57-06-7 | 896 | 16612.29  ±280.57 | 15429.24  ±108.71 | 11459.29  ±80.79 | 10611.25  ±173.74 | 8376.66  ±42.16 | 6501.84  ±48.18 | 4452.94  ±66.17 | 3182.79  ±65.58 | 2370.49  ±53.89 | 1473.15  ±28.37 | 1070.03  ±36.53 | 951.01  ±11.74 | 940.13  ±15.52 | 863.11  ±23.83 | 819.66  ±14.79 | 801.20  ±17.41 | 723.99  ±11.42 |
| E2 | 2-Phenylethyl isothiocyanate | 2257-09-2 | 1457 | 2919.30  ±15.89 | 2256.21  ±18.22 | 1967.38  ±13.26 | 1744.82  ±17.72 | 1592.36  ±18.80 | 1542.83  ±17.89 | 1202.19  ±16.41 | 1172.13  ±21.51 | 1087.63  ±13.26 | 1065.48  ±18.27 | 949.70  ±13.63 | 839.34  ±11.70 | 593.05  ±15.05 | 372.12  ±16.11 | 186.42  ±8.80 | 122.99  ±13.57 | 101.62  ±12.25 |
| E3 | 3-Butenyl isothiocyanate | 3386-97-8 | 983 | 313.55  ±12.56 | 287.20  ±9.35 | 213.93  ±8.73 | 216.22  ±12.76 | 186.22  ±11.08 | 178.32  ±12.28 | 162.22  ±8.80 | 168.06  ±7.38 | 152.24  ±4.26 | 148.23  ±8.93 | 129.56  ±4.76 | 105.81  ±6.33 | 94.17  ±2.78 | 82.05  ±5.11 | 63.25  ±5.69 | 48.37  ±3.98 | 31.41  ±2.92 |
| E4 | 3-(Methylthio)propyl isothiocyanate | 505-79-3 | 1318 | 49.41  ±2.23 | 35.77  ±3.39 | 33.63  ±2.83 | 33.05  ±2.98 | 29.65  ±2.67 | 25.85  ±1.98 | 24.82  ±2.41 | 24.47  ±2.74 | 23.50  ±2.28 | 17.30  ±1.10 | 8.96  ±0.45 | 4.32  ±0.52 | - ^a^ | - | - | - | - |
| E5 | Butyl isothiocyanate | 592-82-5 | 947 | 340.02  ±3.33 | 286.29  ±4.93 | 282.87  ±2.35 | 271.55  ±5.37 | 226.31  ±3.34 | 199.97  ±2.16 | 190.21  ±1.45 | 135.90  ±1.89 | 102.35  ±1.46 | 74.64  ±1.97 | 63.90  ±2.35 | 61.30  ±2.28 | 44.63  ±1.85 | 27.12  ±1.44 | 11.07  ±0.86 | - | - |
| E6 | Isobutyl isothiocyanate | 591-82-2 | 926 | 99.83  ±1.94 | 97.95  ±1.89 | 96.42  ±2.19 | 90.98  ±1.42 | 90.25  ±1.51 | 85.56  1.08 | 59.12  ±1.65 | 33.16  ±0.56 | 12.51  ±1.50 | - | - | - | - | - | - | - | - |
| E7 | 3-Methylbutyl isothiocyanate | 628-03-5 | 1039 | 14.50  ±0.60 | 12.95  ±0.77 | 9.42  ±0.39 | 8.37  ±0.35 | 8.27  ±0.60 | 5.26  ±0.42 | 3.11  ±0.22 | 0.98  ±0.42 | 0.72  ±0.13 | 0.64  ±0.11 | - | - | - | - | - | - | - |
| E8 | Pentyl isothiocyanate | 629-12-9 | 1067 | 8.69  ±0.84 | 7.89  ±0.54 | 4.91  ±0.65 | 2.97  ±0.45 | - | - | - | - | - | - | - | - | - | - | - | - | - |
| E9 | Pentadecanoic acid ethyl ester | 41114-00-5 | 1892 | - | 6.12  ±0.43 | 11.58  ±1.21 | 16.83  ±0.99 | 23.78  ±1.72 | 31.81  ±0.82 | 43.05  ±2.43 | 62.30  ±1.79 | 85.82  ±1.14 | 102.74  ±0.85 | 114.26  ±0.98 | 98.55  ±0.80 | 62.72  ±1.97 | 50.61  ±0.89 | 41.58  ±1.15 | 37.08  ±0.80 | 39.24  ±0.89 |
| E10 | Octanoic acid ethyl ester | 106-32-1 | 1192 | - | - | - | 6.42  ±0.59 | 8.04  ±0.80 | 12.54  ±0.72 | 22.14  ±1.31 | 26.21  1.14 | 30.82  ±1.02 | 40.65  ±1.39 | 45.86  ±1.57 | 41.58  ±0.89 | 48.56  ±1.29 | 76.42  ±1.02 | 82.69  ±0.94 | 85.59  ±0.79 | 90.28  ±1.21 |
| E11 | Tridecanoic acid methyl ester | 1731-88-0 | 1628 | - | - | - | - | - | - | 0.06  ±0.01 | 0.10  ±0.04 | 0.18  ±0.06 | 0.26  ±0.05 | 0.21  ±0.06 | 0.49  ±0.11 | 1.24  ±0.22 | 2.56  ±0.37 | 2.31  ±0.24 | 3.55  ±0.26 | 4.89  ±0.35 |
| E12 | (Z)-9-Octadecenoic acid methyl ester | 112-62-9 | 2097 | - | - | - | 0.25  ±0.05 | 1.45  ±0.08 | 0.49  ±0.05 | 0.18  ±0.04 | - | - | - | - | - | - | - | - | - | - |
| E13 | 9,12-Octadecadienoic acid(Z,Z)-methyl ester | 112-63-0 | 2086 | - | 0.17  ±0.04 | 0.27  ±0.02 | 0.30  ±0.03 | 0.49  ±0.03 | 0.29  ±0.04 | 0.12  ±0.03 | 0.27  ±0.02 | 0.32  ±0.01 | 0.40  ±0.04 | 0.48  ±0.05 | 0.71  ±0.14 | 0.77  ±0.13 | 0.79  ±0.18 | 0.94  ±0.18 | 0.92  ±0.15 | 1.10  ±0.17 |
| **Aldehyde** | |  |  |  |  |  |  |  |  |  |  |  |  |  |  |  |  |  |  |  |
| A1 | Hexanal | 66-25-1 | 809 | - | - | 1.22  ±0.29 | 1.84  ±0.70 | 2.77  ±0.18 | 4.24  ±0.22 | 5.11  ±0.25 | 4.46  ±0.46 | 5.56  ±0.20 | 5.91  ±0.49 | 7.05  ±0.73 | 9.46  ±0.53 | 13.63  ±0.67 | 17.42  ±1.04 | 31.72  ±1.87 | 32.89  ±1.25 | 47.37  ±1.16 |
| A2 | (E)- 2-Hexenal | 6728-26-3 | 859 | 1.04  ±0.10 | 2.57  ±0.35 | 2.88  ±0.19 | 1.56  ±0.36 | 2.46  ±0.29 | 2.91  ±0.49 | 3.75  ±0.45 | 3.57  ±0.34 | 6.88  ±0.41 | 9.73  ±0.38 | 12.86  ±0.53 | 12.75  ±0.46 | 15.89  ±0.55 | 18.28  ±0.98 | 17.37  ±0.62 | 15.68  ±0.58 | 12.56  0.99 |
| A3 | (E)-2-Heptenal | 18829-55-5 | 957 | 2.56  ±0.32 | 2.78  ±0.53 | 2.66  ±0.38 | 2.18  ±0.32 | 1.62  ±0.21 | 1.55  ±0.28 | 0.70  ±0.17 | 0.58  ±0.15 | 1.17  ±0.14 | 1.89  ±0.23 | 2.51  ±0.18 | 2.66  ±0.30 | 2.83  ±0.41 | 4.72  ±0.43 | 6.89  ±0.46 | 7.22  ±0.24 | 8.58  ±0.19 |
| A4 | (E,E)-2,4-Heptadienal | 4313-03-5 | 1008 | 4.96  ±0.33 | 19.37  ±0.64 | 23.32  ±0.93 | 29.52  ±0.76 | 25.47  ±0.95 | 17.53  ±0.65 | 14.38  ±0.96 | 9.18  ±0.37 | 9.79  ±0.46 | 15.90  ±0.55 | 23.41  ±0.83 | 24.48  ±0.74 | 29.42  ±0.79 | 35.18  ±0.86 | 43.13  ±1.67 | 48.33  ±1.52 | 55.06  ±1.39 |
| A5 | Phenylacetaldehyde | 122-78-1 | 1046 | 66.83  ±1.77 | 71.08  ±1.30 | 73.54  ±2.03 | 117.98  ±3.18 | 171.09  ±2.34 | 194.73  ±2.15 | 216.65  ±4.08 | 244.18  ±2.98 | 299.61  ±3.20 | 322.81  ±5.65 | 362.70  ±1.82 | 441.32  ±3.91 | 423.98  ±6.32 | 448.01  ±13.94 | 413.03  ±8.90 | 432.19  ±7.76 | 414.95  ±7.45 |
| A6 | (E)-2-Octenal | 2548-87-0 | 1062 | 11.60  ±0.89 | 15.52  ±1.30 | 14.42  ±1.08 | 11.33  ±0.80 | 10.61  ±0.87 | 9.81  ±0.35 | 5.87  ±0.31 | 4.46  ±0.44 | 3.98  ±0.21 | 3.05  ±0.38 | 2.91  ±0.40 | 2.66  ±0.13 | 8.50  ±0.29 | 14.49  ±0.50 | 20.33  ±0.93 | 25.51  ±1.34 | 28.33  ±1.29 |
| A7 | Nonanal | 124-19-6 | 1106 | 9.85  ±0.51 | 10.50  ±0.62 | 7.24  ±0.39 | 12.36  ±0.81 | 11.55  ±0.70 | 6.38  ±0.56 | 4.82  ±0.43 | 6.83  ±0.57 | 8.62  ±0.45 | 10.19  ±0.66 | 12.57  ±0.41 | 13.53  ±0.30 | 13.90  ±0.35 | 14.80  ±0.38 | 18.07  ±0.71 | 21.55  ±0.84 | 24.50  ±0.91 |
| A8 | (E,E)-2,4-Decadienal | 25152-84-5 | 1343 | 4.21  ±0.27 | 4.38  ±0.21 | 4.70  ±0.35 | 3.59  ±0.29 | 5.90  ±0.61 | 8.62  ±0.58 | 9.05  ±0.48 | 7.97  ±0.83 | 6.60  ±0.29 | 4.53  ±0.34 | 2.05  ±0.29 | 1.74  ±0.21 | 0.71  ±0.13 | 0.54  ±0.09 | - | - | - |
| A9 | (E,Z)- 2,6-Nonadienal | 557-48-2 | 1157 | 8.86  ±0.36 | 13.88  ±0.70 | 17.78  ±0.94 | 15.95  ±0.55 | 14.33  ±0.76 | 12.49  ±0.55 | 9.53  ±0.74 | 10.77  ±0.44 | 16.05  ±0.69 | 21.19  ±0.87 | 26.47  ±0.96 | 17.42  ±0.55 | 16.46  ±0.60 | 15.73  ±0.41 | 12.22  ±0.37 | 11.24  ±0.77 | 11.75  ±0.93 |
| A10 | 4-Ethylbenzaldehyde | 4748-78-1 | 1179 | 4.69  ±0.29 | 4.86  ±0.21 | 3.94  ±0.26 | 4.25  ±0.24 | 5.03  ±0.34 | 5.57  ±0.17 | 6.90  ±0.57 | 6.05  ±0.38 | 6.42  ±0.32 | 6.55  ±0.37 | 7.44  ±0.28 | 6.93  ±0.48 | 7.18  ±0.35 | 7.47  ±0.38 | 7.52  ±0.30 | 7.74  ±0.46 | 8.02  ±0.44 |
| A11 | 2-Undecenal | 2463-77-6 | 1366 | 0.41  ±0.09 | 0.57  ±0.10 | 0.64  ±0.11 | 0.53  ±0.09 | 1.09  ±0.20 | 1.43  ±0.27 | 1.52  ±0.16 | 2.91  ±0.33 | 4.63  ±0.52 | 5.83  ±0.60 | 6.65  ±0.43 | 5.08  ±0.33 | 3.87  ±0.35 | 3.03  ±0.17 | 4.28  ±0.28 | 3.97  ±0.23 | 3.07  ±0.21 |
| **Alcohols** | |  |  |  |  |  |  |  |  |  |  |  |  |  |  |  |  |  |  |  |
| L1 | 3-Methyl-1-butanol | 123-51-3 | 751 | - | - | - | - | 64.13  ±2.08 | 34.96  ±1.67 | 28.92  ±0.74 | 6.05  ±0.57 | - | - | - | - | - | - | - | - | - |
| L2 | (E)-2-Octen-1-ol | 18409-17-1 | 1073 | - | - | 12.51  ±1.16 | 33.78  ±2.21 | 74.13  ±1.64 | 93.22  ±2.14 | 131.54  ±1.95 | 102.70  ±2.23 | 91.24  ±1.80 | 63.69  ±2.09 | 75.09  ±1.51 | 53.94  ±1.64 | 36.42  ±1.03 | 24.49  ±1.14 | 43.83  ±1.67 | 40.80  ±1.85 | 44.15  ±1.56 |
| L3 | 1-Octanol | 111-87-5 | 1079 | - | - | - | 12.70  ±1.05 | 11.50  ±0.98 | - | - | - | - | - | 13.03  ±1.65 | 15.50  ±1.26 | 15.80  ±0.82 | 19.50  ±0.97 | 34.47  ±1.11 | 36.38  ±1.07 | 47.28  ±1.02 |
| L4 | Phenylethyl alcohol | 60-12-8 | 1132 | - | - | - | 6.40  ±0.30 | 19.50  ±0.84 | 21.89  ±0.94 | 24.47  ±0.89 | 22.79  ±1.63 | 34.36  ±1.37 | 47.07  ±1.20 | 41.42  ±0.84 | 26.35  ±1.04 | 35.26  ±1.18 | 41.47  ±0.95 | 57.48  ±0.80 | 59.87  ±1.93 | 76.19  ±1.54 |
| **Ketones** | |  |  |  |  |  |  |  |  |  |  |  |  |  |  |  |  |  |  |  |
| K1 | 3,5-Octadien-2-one | 38284-27-4 | 1098 | - | - | - | - | 21.79  ±1.06 | 32.66  ±0.83 | 38.02  ±1.32 | 47.51  ±0.92 | 63.96  ±1.58 | 76.28  ±1.63 | 86.62  ±1.96 | 92.34  ±1.24 | 99.34  ±0.91 | 106.59  ±0.82 | 118.03  ±1.08 | 142.78  ±1.17 | 168.94  ±1.24 |
| K2 | 6,10-Dimethyl-5,9-undecadien-2-one | 689-67-8 | 1462 | 6.93  ±0.47 | 8.33  ±0.21 | 9.42  ±0.28 | 11.90  ±0.57 | 12.86  ±0.41 | 13.49  ±0.93 | 14.53  ±1.05 | 15.61  ±0.79 | 15.60  ±0.63 | 16.60  ±0.86 | 18.76  ±0.45 | 21.12  ±1.03 | 23.49  ±1.15 | 24.32  ±0.98 | 25.74  ±1.10 | 26.02  ±0.52 | 28.39  ±0.96 |
| **Acids** | |  |  |  |  |  |  |  |  |  |  |  |  |  |  |  |  |  |  |  |
| C1 | Octanoic acid | 124-07-2 | 1187 | 20.22  ±1.87 | 19.03  ±1.28 | 8.53  ±0.39 | - | - | - | - | 13.22  ±1.83 | 20.95  ±1.35 | 32.27  ±1.08 | 36.48  ±0.92 | 40.84  ±0.97 | 48.89  ±1.13 | 57.35  ±0.92 | 80.57  ±0.98 | 97.03  ±1.54 | 73.24  ±1.71 |
| C2 | Linolenic acid | 463-40-1 | 2153 | 9.08  ±0.59 | 0.72  ±0.07 | 0.53  ±0.03 | - | - | - | - | - | - | - | - | - | - | - | - | - | - |
| **Nitriles** | |  |  |  |  |  |  |  |  |  |  |  |  |  |  |  |  |  |  |  |
| N1 | 3-Butenenitrile | 109-75-1 | 658 | 26.07  ±0.59 | - | - | - | - | - | - | - | - | 22.52  ±1.08 | 34.07  ±1.77 | 42.54  ±1.18 | 52.13  ±1.45 | 58.62  ±1.20 | 64.93  ±1.23 | 74.17  ±1.70 | 78.45  ±0.92 |
| N2 | Benzenepropanenitrile | 645-59-0 | 1247 | 23.85  ±1.94 | 27.46  ±1.04 | 38.65  ±1.17 | 16.85  ±0.95 | 22.08  ±1.46 | 23.82  ±1.66 | 43.52  ±2.12 | 69.30  ±1.88 | 72.73  ±1.65 | 76.07  ±1.17 | 79.98  ±1.46 | 84.67  ±1.20 | 89.98  ±1.48 | 93.63  ±1.33 | 96.87  ±1.47 | 93.86  ±1.55 | 87.40  ±1.09 |
| **Others** | |  |  |  |  |  |  |  |  |  |  |  |  |  |  |  |  |  |  |  |
| O1 | 2-Ethylfuran | 3208-16-0 | 706 | 26.46  ±1.15 | 12.64  ±1.29 | 15.85  ±1.08 | 18.44  ±1.12 | 30.05  ±1.41 | 32.29  ±1.18 | 41.73  ±1.76 | 43.06  ±1.25 | 46.96  ±1.75 | 49.89  ±1.44 | 52.01  ±1.50 | 47.68  ±1.29 | 45.19  ±1.68 | 42.95  ±0.57 | 42.18  ±1.84 | 38.64  ±1.36 | 35.89  ±1.76 |
| O2 | 2-Pentylfuran | 3777-69-3 | 1003 | 2.93  ±0.08 | 2.75  ±0.09 | 2.15  ±0.09 | 3.41  ±0.16 | 4.01  ±0.14 | 4.56  ±0.12 | 4.44  ±0.12 | 4.86  ±0.09 | 5.50  ±0.14 | 5.33  ±0.13 | 5.18  ±0.22 | 5.78  ±0.13 | 4.93  ±0.11 | 3.83  ±0.10 | 3.18  ±0.16 | 2.55  ±0.08 | 2.22  ±0.14 |
| O3 | (Z)-2-(2-Pentenyl) furan | 70424-13-4 | 1015 | - | 5.04  ±0.27 | 3.35  ±0.17 | 4.66  ±0.34 | 4.41  ±0.17 | 3.39  ±0.45 | 2.46  ±0.38 | 1.45  ±0.29 | 2.60  ±0.39 | 3.47  ±0.46 | 3.84  ±0.34 | 5.42  ±0.36 | 6.27  ±0.28 | 7.51  ±0.44 | 8.05  ±0.39 | 8.91  ±0.63 | 10.39  ±1.08 |
| O4 | Dimethyl trisulfide | 3658-80-8 | 975 | - | 10.35  ±1.04 | 11.60  ±0.77 | 7.94  ±0.51 | 8.84  ±0.71 | 10.42  ±1.01 | 11.62  ±0.72 | 13.61  ±1.24 | 15.27  ±1.09 | 21.75  ±0.83 | 25.79  ±1.21 | 22.97  ±1.46 | 24.62  ±1.21 | 25.69  ±1.19 | 26.33  ±0.87 | 27.70  ±0.83 | 28.90  ±0.94 |

a: "-", Undetected
