# Supplementary material for: Analysis of the microbial community structure and flavor components succession during salt‐reducing pickling process of zhacai (preserved mustard tuber)
Source: Food Sci Nutr. 2023 Apr 17;11(6):3154–70. doi: 10.1002/fsn3.3297 (PMC10261794; doi:10.1002/fsn3.3297)
Supplement: Supplementary file 1 — Appendix S1. [file FSN3-11-3154-s001.zip › ═╝║═▒φ/S3 Table. Amino acid concentrations during the zhacai pickling process.docx]

S3 Table. Amino acid concentrations during the *zhacai* pickling process

| Kinds of amino acids | | Concentration of amino acids（mg·kg ^-1^） | | | | | | | | | | | | | | | | |
| --- | --- | --- | --- | --- | --- | --- | --- | --- | --- | --- | --- | --- | --- | --- | --- | --- | --- | --- |
|  |  | S0 | S11 | S12 | S21 | S22 | S23 | S24 | S31 | S32 | S33 | S34 | S41 | S42 | S43 | S44 | S45 | S46 |
| Umami | L-Aspartic acid (Asp) | 37.11±  0.24 | 32.92±  0.34 | 31.62±  0.29 | 38.36±  0.39 | 34.61±  0.29 | 51.64±  0.45 | 64.55±  0.38 | 68.53±  0.42 | 73.43±  0.49 | 91.98±  0.58 | 107.08±  0.45 | 102.41±  0.62 | 126.19±  0.81 | 132.43±  0.86 | 146.57±  0.56 | 164.48±  0.57 | 192.73±  0.65 |
|  | L-Glutamic acid (Glu) | 164.84±  0.67 | 182.32±  0.79 | 221.53±  0.92 | 267.85±0.68 | 299.74±0.67 | 348.13±0.43 | 535.06±0.36 | 559.04±0.48 | 617.44±0.74 | 656.83±0.86 | 742.91±0.58 | 720.76±0.62 | 813.59±0.88 | 860.20±0.56 | 881.84±  0.75 | 979.77±0.56 | 1091.93  ±0.66 |
| Sweet | Glycine (Gly) | 180.94±  0.33 | 149.34±  0.26 | 133.32±  0.25 | 160.69±  0.47 | 217.65±  0.58 | 247.68±  0.53 | 291.35±  1.14 | 323.18±  0.71 | 290.38±  0.68 | 324.32±  0.55 | 329.57±  0.55 | 339.04±  0.64 | 383.39±1.20 | 431.49±0.99 | 447.31±  0.61 | 500.44±  0.75 | 546.45±  1.00 |
|  | L-Alanine (Ala) | 477.42±  1.28 | 437.97±  0.69 | 494.36±0.85 | 627.48±  0.81 | 766.38±  1.10 | 974.04±0.87 | 1060.84±1.09 | 1100.55±0.78 | 1104.61±0.87 | 1172.51±1.03 | 1127.37±0.87 | 1180.45±0.89 | 1245.44±0.79 | 1217.64±0.69 | 1303.98±1.34 | 1379.97±0.98 | 1360.11±0.93 |
|  | L-Serine (Ser) | 120.36±  0.76 | 152.18±  0.84 | 158.31±0.79 | 126.81±  0.65 | 179.10±  0.79 | 254.57±  1.17 | 274.61±  0.90 | 316.50±  0.97 | 387.29±  0.95 | 398.29±  0.65 | 411.22±  0.58 | 442.07±  0.76 | 457.39±  0.57 | 463.72±  0.41 | 483.30±  0.53 | 495.49±  0.94 | 474.23±  0.35 |
|  | L-Threonine (Thr) | 248.07±  0.35 | 191.85±  0.66 | 214.39±  0.75 | 203.06±  0.38 | 234.75±  0.55 | 290.65±  0.96 | 263.72±  0.82 | 281.15±  0.47 | 318.53±  0.33 | 304.13±  0.37 | 331.06±  0.56 | 354.74±  0.43 | 338.53±  0.35 | 359.57±  0.62 | 368.18±  0.70 | 372.46±  0.61 | 358.61±  0.69 |
| Aromatic amino acids | L-Tyrosine (Tyr) | 170.40±  1.31 | 133.90±  0.58 | 101.99±  0.76 | 90.68±  0.87 | 117.59±  0.77 | 143.56±  0.61 | 167.61±  0.51 | 144.60±  0.67 | 126.87±  0.76 | 112.84±  0.58 | 101.12±  0.70 | 92.27±  0.55 | 91.84±  0.92 | 89.72±  0.97 | 89.41±  0.66 | 85.16±  0.74 | 82.10±  0.65 |
|  | L-Phenylalanine (Phe) | 82.19±  0.83 | 101.92±0.73 | 138.44±0.93 | 139.53±  1.15 | 164.26±0.74 | 192.81±1.06 | 210.81±  1.04 | 191.17±  1.12 | 207.63±  1.33 | 230.85±  0.93 | 260.36±  0.94 | 218.32±  0.85 | 234.75±  1.06 | 252.76±  0.76 | 235.06±  0.50 | 215.17±  0.61 | 211.72±  0.52 |
|  | L-Tryptophan (Trp) | 22.81±  0.87 | 23.96±  0.61 | 27.68±  0.47 | 23.64±  0.27 | 28.44±  0.35 | 34.18±  0.26 | 39.43±  0.38 | 42.55±  0.31 | 46.60±  0.30 | 49.86±  0.38 | 50.57±  0.32 | 57.76±  0.35 | 66.74±  0.27 | 77.34±  0.25 | 84.55±  0.22 | 84.92±  0.26 | 85.11±  0.18 |
| Bitter | L-Histidine (His) | 27.42±  0.39 | 22.52±  0.36 | 21.38±  0.35 | 31.66±  0.46 | 42.51±  0.31 | 67.94±  0.55 | 69.16±  0.28 | 83.81±  0.48 | 96.74±  0.44 | 86.89±  0.51 | 89.36±  0.29 | 84.95±  0.61 | 76.49±  0.37 | 77.65±  0.70 | 75.53±  0.43 | 71.69±  0.75 | 67.49±  0.69 |
|  | L-Arginine (Arg) | 214.05±  0.37 | 228.20±  0.41 | 258.48±  0.39 | 275.47±  0.36 | 299.44±  0.77 | 323.55±  0.60 | 369.90±  0.54 | 393.73±  0.50 | 319.50±  0.29 | 346.67±  0.51 | 368.64±  0.55 | 328.52±  0.44 | 374.73±  0.49 | 397.27±  0.59 | 398.50±  0.82 | 432.90±  0.67 | 539.92±0.75 |
|  | L-Valine (Val) | 215.83±  0.59 | 273.23±  0.55 | 287.36±  0.46 | 229.03±  0.43 | 294.58±  0.47 | 371.79±  0.59 | 341.41±  0.36 | 381.49±  0.86 | 393.18±  0.65 | 343.26±  0.43 | 393.97±  0.34 | 412.27±  0.73 | 353.95±  0.32 | 379.17±  0.34 | 343.25±  0.61 | 392.78±  0.64 | 351.08±  0.39 |
|  | L-Leucine (Leu) | 202.59±  0.82 | 247.71±  0.52 | 276.29±  0.68 | 218.11±  0.45 | 273.85±  0.53 | 361.95±0.58 | 350.89±  0.46 | 390.48±  0.86 | 415.50±  0.93 | 344.81±  0.60 | 301.13±  0.42 | 331.25±  0.57 | 377.32±  0.75 | 382.27±  0.56 | 361.09±  0.58 | 418.07±  0.66 | 414.12±  0.74 |
|  | L-Isoleucine (Ile) | 164.22±  0.32 | 159.91±  0.80 | 192.59±  0.46 | 179.73±  0.52 | 199.06±  0.73 | 247.94±  0.41 | 243.84±  0.47 | 240.66±  0.36 | 267.88±  0.34 | 319.79±  0.54 | 342.67±  0.47 | 279.12±  0.76 | 248.93±  0.48 | 228.71±  0.43 | 242.72±  0.84 | 268.89±  0.47 | 271.27±  0.66 |
|  | L-Methionine (Met) | 81.64±  0.70 | 87.20±  0.64 | 89.23±  0.76 | 72.76±  0.69 | 85.04±  0.62 | 102.80±  0.52 | 109.31±  0.61 | 110.21±0.57 | 117.81±  0.66 | 129.34±  0.46 | 134.23±  0.43 | 117.69±  0.57 | 104.52±  0.42 | 102.30±  0.39 | 97.79±  0.78 | 93.84±  0.65 | 91.32±  0.40 |
| Other amino acids | L-Lysine (Lys) | 218.64±  0.93 | 245.51±  1.26 | 265.47±  0.76 | 221.46±  1.08 | 268.52±  1.03 | 350.27±  1.45 | 387.94±  1.26 | 438.79±  1.32 | 496.36±  1.06 | 538.34±  0.95 | 578.41±  1.28 | 621.91±  0.68 | 674.47±  1.23 | 717.55±0.96 | 745.44±  1.29 | 802.55±0.86 | 849.86±0.52 |
|  | L-Proline (Pro) | 290.53±  1.23 | 349.46±  0.83 | 380.07±0.99 | 351.66±1.09 | 391.55±0.94 | 421.70±  0.89 | 434.36±  0.79 | 440.58±  1.13 | 469.18±  1.42 | 495.56±  1.12 | 525.57±  0.78 | 571.64±  1.19 | 502.73±  0.84 | 477.92±  0.89 | 441.65±  0.71 | 408.70±  0.79 | 404.45±0.95 |
|  | 4-Aminobutyric acid (GABA) | 1350.71±4.58 | 1402.05±4.40 | 1416.45±3.57 | 1368.19±5.64 | 1482.12±5.21 | 1532.40±4.94 | 1544.53±3.58 | 1694.15±4.16 | 1747.14±4.90 | 1757.63±4.72 | 1785.06±4.82 | 1862.16±3.58 | 1964.46±2.89 | 2089.24±5.15 | 2218.70±4.57 | 2356.25±4.89 | 2767.48±3.83 |
|  | L-Asparagine (Asn) | 236.45±  0.69 | 241.66±  0.74 | 246.41±  0.65 | 260.64±  0.92 | 279.16±  0.84 | 294.93±  0.45 | 334.57±  0.80 | 336.95±  0.30 | 381.21±  0.45 | 419.20±0.41 | 423.26±  0.73 | 437.42±  0.87 | 467.32±  0.98 | 475.35±  0.51 | 480.69±0.92 | 528.27±  0.39 | 488.63±  0.70 |
|  | L-Ornithine hydrochloride (Orn) | 12.44±  0.42 | 16.64±  0.28 | 20.62±  0.41 | 24.57±  0.29 | 25.39±  0.53 | 26.06±  0.19 | 27.33±  0.25 | 28.30±  0.33 | 28.56±  0.47 | 31.41±  0.36 | 31.96±  0.76 | 37.74±  0.32 | 49.88±  0.23 | 55.90±  0.32 | 61.19±  0.29 | 66.45±  0.38 | 77.18±  0.36 |
|  | L-Glutamine (Gln) | 6369.09±3.85 | 6380.64±1.97 | 6500.56±3.32 | 8482.35±3.88 | 9265.42±2.11 | 9583.24±1.93 | 9660.80±2.80 | 10351.67±2.92 | 10584.15±2.74 | 10992.65±2.68 | 11320.27±2.49 | 11190.73±2.95 | 11873.19±2.50 | 11986.92±2.33 | 12326.39±1.84 | 12450.91±2.46 | 12171.89±2.65 |
