# Supplementary material for: Analysis of the microbial community structure and flavor components succession during salt‐reducing pickling process of zhacai (preserved mustard tuber)
Source: Food Sci Nutr. 2023 Apr 17;11(6):3154–70. doi: 10.1002/fsn3.3297 (PMC10261794; doi:10.1002/fsn3.3297)
Supplement: Supplementary file 1 — Appendix S1. [file FSN3-11-3154-s001.zip › ═╝║═▒φ/S4 Table. PacBio Sequel sequencing data for analysis of bacterial diversity including a┴ diversity indices.docx]

# S4 Table. PacBio Sequel sequencing data for analysis of bacterial diversity including α diversity indices

| Group | Sample | Nonsingleton | α diversity indices | | | | ASV number | Domain | Phylum | Class | Order | Family | Genus |
| --- | --- | --- | --- | --- | --- | --- | --- | --- | --- | --- | --- | --- | --- |
|  |  |  | Shannon | Simpson | Chao1 | Goods coverage |  |  |  |  |  |  |  |
| Raw material | S0 | 3616 | 3.976 | 0.849 | 127.143 | 0.9933 | 121 | 1 | 4 | 6 | 12 | 19 | 16 |
| First stage | S11 | 6465 | 4.531 | 0.878 | 209.263 | 0.9847 | 195 | 1 | 8 | 17 | 31 | 61 | 77 |
|  | S12 | 4789 | 4.572 | 0.875 | 271.054 | 0.9779 | 210 | 1 | 6 | 12 | 26 | 53 | 79 |
| Second stage | S21 | 4534 | 4.285 | 0.815 | 326.392 | 0.9714 | 254 | 1 | 6 | 12 | 25 | 53 | 79 |
|  | S22 | 4705 | 2.369 | 0.492 | 202.389 | 0.9824 | 148 | 1 | 5 | 9 | 22 | 36 | 50 |
|  | S23 | 4344 | 4.848 | 0.874 | 302.49 | 0.9757 | 244 | 1 | 7 | 14 | 26 | 60 | 94 |
|  | S24 | 7140 | 4.360 | 0.912 | 167.663 | 0.9878 | 158 | 1 | 6 | 9 | 20 | 42 | 61 |
| Third stage | S31 | 4496 | 3.271 | 0.690 | 281.872 | 0.9750 | 196 | 1 | 6 | 10 | 22 | 46 | 59 |
|  | S32 | 5565 | 4.444 | 0.861 | 302.476 | 0.9747 | 249 | 1 | 8 | 14 | 29 | 58 | 84 |
|  | S33 | 3850 | 3.458 | 0.776 | 225.953 | 0.9797 | 164 | 1 | 5 | 12 | 23 | 44 | 57 |
|  | S34 | 4998 | 4.500 | 0.888 | 310.417 | 0.9719 | 258 | 1 | 8 | 16 | 30 | 60 | 76 |
| Fourth stage | S41 | 6594 | 2.818 | 0.819 | 89.5027 | 0.9928 | 72 | 1 | 4 | 9 | 17 | 25 | 35 |
|  | S42 | 3450 | 5.166 | 0.938 | 220.962 | 0.9850 | 172 | 1 | 4 | 9 | 18 | 33 | 38 |
|  | S43 | 5579 | 2.582 | 0.687 | 118.539 | 0.9901 | 98 | 1 | 4 | 8 | 18 | 31 | 38 |
|  | S44 | 3759 | 3.345 | 0.849 | 183.496 | 0.9846 | 101 | 1 | 5 | 8 | 14 | 23 | 31 |
|  | S45 | 5384 | 3.574 | 0.842 | 171.931 | 0.9852 | 140 | 1 | 5 | 11 | 22 | 38 | 53 |
|  | S46 | 4858 | 3.404 | 0.799 | 169.374 | 0.9871 | 122 | 1 | 3 | 8 | 16 | 31 | 49 |
