# Supplementary material for: Analysis of the microbial community structure and flavor components succession during salt‐reducing pickling process of zhacai (preserved mustard tuber)
Source: Food Sci Nutr. 2023 Apr 17;11(6):3154–70. doi: 10.1002/fsn3.3297 (PMC10261794; doi:10.1002/fsn3.3297)
Supplement: Supplementary file 1 — Appendix S1. [file FSN3-11-3154-s001.zip › ═╝║═▒φ/S5 Table. PacBio Sequel sequencing data for analysis of fungal diversity including a┴ diversity indices.docx]

# S5 Table. PacBio Sequel sequencing data for analysis of fungal diversity including α diversity indices

| Group | Sample | Nonsingleton | α diversity indices | | | | ASV number | Domain | Phylum | Class | Order | Family | Genus |
| --- | --- | --- | --- | --- | --- | --- | --- | --- | --- | --- | --- | --- | --- |
|  |  |  | Shannon | Simpson | Chao1 | Goods coverage |  |  |  |  |  |  |  |
| Raw material | S0 | 4847 | 3.249 | 0.796 | 81.729 | 0.9966 | 70 | 1 | 2 | 9 | 16 | 21 | 23 |
| First stage | S11 | 4756 | 3.070 | 0.789 | 61.421 | 0.9970 | 53 | 1 | 2 | 6 | 9 | 16 | 16 |
|  | S12 | 4192 | 3.543 | 0.822 | 75.312 | 0.9969 | 69 | 1 | 2 | 7 | 10 | 12 | 15 |
| Second stage | S21 | 5392 | 1.398 | 0.503 | 48.859 | 0.9965 | 41 | 1 | 2 | 6 | 9 | 13 | 14 |
|  | S22 | 5227 | 0.966 | 0.238 | 25.891 | 0.9991 | 25 | 1 | 2 | 6 | 7 | 10 | 10 |
|  | S23 | 4389 | 3.325 | 0.829 | 99.083 | 0.9948 | 81 | 1 | 2 | 9 | 14 | 21 | 26 |
|  | S24 | 4903 | 1.125 | 0.338 | 25.567 | 0.9987 | 21 | 1 | 2 | 6 | 6 | 8 | 9 |
| Third stage | S31 | 4578 | 3.037 | 0.693 | 87.579 | 0.9957 | 79 | 1 | 2 | 9 | 14 | 21 | 25 |
|  | S32 | 5657 | 1.094 | 0.293 | 57.823 | 0.9963 | 39 | 1 | 2 | 8 | 11 | 16 | 18 |
|  | S33 | 6333 | 0.696 | 0.166 | 37.173 | 0.9977 | 32 | 1 | 2 | 6 | 6 | 10 | 12 |
|  | S34 | 5911 | 1.009 | 0.289 | 37.105 | 0.9978 | 34 | 1 | 2 | 6 | 11 | 16 | 17 |
| Fourth stage | S41 | 4746 | 2.078 | 0.636 | 35.660 | 0.9987 | 34 | 1 | 2 | 7 | 9 | 13 | 14 |
|  | S42 | 5013 | 0.823 | 0.218 | 37.961 | 0.9977 | 33 | 1 | 2 | 7 | 10 | 13 | 14 |
|  | S43 | 12913 | 1.666 | 0.560 | 47.915 | 0.9966 | 47 | 1 | 2 | 8 | 10 | 14 | 17 |
|  | S44 | 5822 | 0.614 | 0.226 | 16.475 | 0.9988 | 14 | 1 | 2 | 3 | 3 | 5 | 6 |
|  | S45 | 5377 | 1.578 | 0.481 | 29.416 | 0.9988 | 29 | 1 | 2 | 9 | 11 | 14 | 14 |
|  | S46 | 8742 | 1.242 | 0.462 | 29.620 | 0.9985 | 27 | 1 | 2 | 6 | 8 | 10 | 13 |
