# Supplementary material for: Analysis of the microbial community structure and flavor components succession during salt‐reducing pickling process of zhacai (preserved mustard tuber)
Source: Food Sci Nutr. 2023 Apr 17;11(6):3154–70. doi: 10.1002/fsn3.3297 (PMC10261794; doi:10.1002/fsn3.3297)
Supplement: Supplementary file 1 — Appendix S1. [file FSN3-11-3154-s001.zip › ═╝║═▒φ/Table 1 The information, basic chemical indices and organic acid concentrations.docx]

Table 1 The information, basic chemical indices, organic acid and monosaccharide concentrations in *zhacai* samples during the pickling process ^a, b^

| Pickling stage | Sample | Pickling  days | Titratable acidity ^c^  (g·kg^-1^) | NaCl concentration (g·kg^-1^) | Nitrite concentration (mg·kg^-1^) | Organic acid concentration (g·kg^-1^) | | | | | Monosaccharide concentration (μg·kg^-1^) | | | |
| --- | --- | --- | --- | --- | --- | --- | --- | --- | --- | --- | --- | --- | --- | --- |
|  |  |  |  |  |  | Oxalic acid | Malic acid | Lactic acid | Acetic acid | Succinic acid | Glucose | Fructose | Galactose | Total |
| Raw material | S0 | 0 | 0.77±0.06 ^o^ | 5.06±0.17 ^q^ | 0.48±0.012 ^i^ | 0.55±0.04 ^h^ | 0.34±0.05 ^i^ | 0.69±0.05 ^l^ | 0.39±0.04 ^i^ | 2.58±0.11 ^l^ | 9.33±0.36 ^a^ | 9.26±0.41 ^a^ | 0.02±0.01 ^i^ | 18.97±0.76 ^a^ |
| First | S11 | 4 | 1.81±0.07 ^n^ | 13.85±0.10 ^p^ | 0.88±0.011 ^de^ | 0.73±0.09 ^efg^ | 0.53±0.06 ^h^ | 3.65±0.15 ^k^ | 0.71±0.06 ^h^ | 2.84±0.07 ^k^ | 8.12±0.23 ^b^ | 8.45±0.54 ^ab^ | 0.05±0.01 ^i^ | 16.88±0.79 ^ab^ |
|  | S12 | 7 | 3.22±0.05 ^m^ | 20.75±0.11 ^o^ | 0.79±0.046 ^fg^ | 0.83±0.06 ^abcde^ | 0.62±0.09 ^h^ | 5.41±0.10 ^j^ | 1.07±0.08 ^g^ | 3.14±0.11 ^j^ | 8.62±0.46 ^ab^ | 7.57±0.56 ^bc^ | 0.06±0.01 ^i^ | 16.50±1.05 ^bc^ |
| Second | S21 | 4 | 3.43±0.13 ^l^ | 22.41±0.09 ^n^ | 0.95±0.046 ^cd^ | 0.84±0.08 ^abcd^ | 0.68±0.04 ^gh^ | 5.82±0.09 ^i^ | 1.18±0.10 ^g^ | 3.49±0.04 ^i^ | 7.13±0.17 ^c^ | 7.34±0.64 ^bcd^ | 0.14±0.02 ^i^ | 14.83±0.63 ^bcd^ |
|  | S22 | 10 | 4.48±0.10 ^k^ | 26.64±0.08 ^m^ | 1.10±0.048 ^b^ | 0.86±0.07 ^abc^ | 0.82±0.08 ^fg^ | 8.25±0.16 ^h^ | 1.88±0.08 ^f^ | 4.24±0.20 ^h^ | 6.92±0.64 ^c^ | 7.16±0.42 ^cd^ | 0.38±0.05 ^h^ | 14.75±1.15 ^cd^ |
|  | S23 | 16 | 5.33±0.08 ^j^ | 28.16±0.09 ^l^ | 0.87±0.048 ^def^ | 0.81±0.07 ^bcdef^ | 0.89±0.04 ^f^ | 10.22±0.12 ^g^ | 2.19±0.12 ^de^ | 4.84±0.13 ^g^ | 5.89±0.56 ^d^ | 7.34±0.47 ^bcd^ | 0.62±0.03 ^g^ | 14.43±1.12 ^cde^ |
|  | S24 | 20 | 6.19±0.05 ^i^ | 31.17±0.10 ^k^ | 0.96±0.048 ^c^ | 0.76±0.05 ^cdefg^ | 0.93±0.03 ^f^ | 11.62±0.14 ^f^ | 2.02±0.07 ^ef^ | 5.26±0.08 ^f^ | 5.17±0.23 ^def^ | 7.40±0.64 ^bcd^ | 0.81±0.04 ^g^ | 14.25±1.01 ^de^ |
| Third | S31 | 2 | 6.07±0.08 ^i^ | 35.81±0.14 ^j^ | 0.75±0.038 ^gh^ | 0.74±0.05 ^defg^ | 0.87±0.04 ^f^ | 11.84±0.09 ^f^ | 1.97±0.04 ^f^ | 5.01±0.03 ^g^ | 5.59±0.32 ^d^ | 6.89±0.67 ^cde^ | 1.07±0.12 ^f^ | 14.41±1.23 ^cde^ |
|  | S32 | 10 | 6.79±0.06 ^h^ | 49.63±0.18 ^i^ | 0.73±0.017 ^gh^ | 0.78±0.02 ^bcdefg^ | 0.94±0.03 ^ef^ | 12.77±0.26 ^e^ | 2.37±0.11 ^d^ | 5.73±0.09 ^cde^ | 5.36±0.52 ^de^ | 6.39±0.57 ^def^ | 1.29±0.15 ^ef^ | 13.92±0.67 ^def^ |
|  | S33 | 20 | 7.29±0.04 ^g^ | 57.32±0.24 ^h^ | 0.85±0.035 ^ef^ | 0.88±0.04 ^ab^ | 1.12±0.07 ^cd^ | 13.09±0.49 ^de^ | 2.91±0.14 ^c^ | 6.23±0.08 ^b^ | 4.78±0.60 ^efg^ | 5.79±0.74 ^efg^ | 1.45±0.12 ^e^ | 12.88±1.50 ^defg^ |
|  | S34 | 37 | 8.07±0.05 ^f^ | 66.71±0.16 ^g^ | 0.71±0.035 ^h^ | 0.93±0.02 ^a^ | 1.41±0.16 ^a^ | 13.42±0.08 ^cd^ | 2.75±0.09 ^c^ | 6.54±0.14 ^a^ | 4.25±0.45 ^g^ | 5.88±0.86 ^efg^ | 1.76±0.13 ^d^ | 12.89±1.52 ^defg^ |
| Fourth | S41 | 5 | 8.10±0.06 ^f^ | 70.49±0.13 ^f^ | 0.88±0.012 ^cde^ | 0.84±0.05 ^abcd^ | 1.33±0.07 ^a^ | 13.70±0.15 ^bc^ | 2.94±0.10 ^c^ | 6.17±0.07 ^b^ | 4.03±0.19 ^gh^ | 5.42±0.64 ^fgh^ | 1.94±0.16 ^d^ | 12.47±1.07 ^efg^ |
|  | S42 | 10 | 8.28±0.03 ^e^ | 76.67±0.20 ^e^ | 1.26±0.057 ^a^ | 0.82±0.02 ^bcdef^ | 1.14±0.04 ^bc^ | 14.04±0.16 ^ab^ | 3.22±0.07 ^b^ | 5.93±0.12 ^c^ | 4.38±0.53 ^fg^ | 5.28±0.42 ^fgh^ | 2.19±0.14 ^c^ | 12.95±1.17 ^defg^ |
|  | S43 | 15 | 8.47±0.07 ^d^ | 81.19±0.12 ^d^ | 0.90±0.012 ^cde^ | 0.76±0.04 ^defg^ | 1.28±0.12 ^ab^ | 14.20±0.08 ^a^ | 3.49±0.20 ^a^ | 5.72±0.13 ^cde^ | 3.23±0.19 ^i^ | 5.67±0.33 ^fg^ | 2.24±0.09 ^bc^ | 12.39±0.50 ^efg^ |
|  | S44 | 21 | 8.69±0.08 ^c^ | 83.72±0.14 ^c^ | 1.10±0.058 ^b^ | 0.73±0.02 ^efg^ | 1.18±0.11 ^bc^ | 14.31±0.09 ^a^ | 3.26±0.12 ^b^ | 5.84±0.10 ^cd^ | 3.12±0.13 ^i^ | 4.92±0.66 ^ghi^ | 2.49±0.15 ^a^ | 11.90±1.03 ^fg^ |
|  | S45 | 28 | 8.94±0.03 ^b^ | 85.47±0.10 ^b^ | 1.10±0.050 ^b^ | 0.72±0.05 ^fg^ | 1.09±0.03 ^cde^ | 14.23±0.10 ^a^ | 3.30±0.06 ^ab^ | 5.63±0.12 ^de^ | 3.08±0.18 ^i^ | 4.10±0.43 ^i^ | 2.46±0.19 ^ab^ | 11.09±0.84 ^g^ |
|  | S46 | 36 | 9.31±0.06 ^a^ | 87.15±0.06 ^a^ | 0.94±0.031 ^cd^ | 0.70±0.02 ^g^ | 0.97±0.09 ^def^ | 14.05±0.07 ^ab^ | 3.17±0.09 ^b^ | 5.57±0.13 ^e^ | 3.36±0.20 ^hi^ | 4.35±0.38 ^hi^ | 2.33±0.19 ^abc^ | 11.53±0.67 ^g^ |

^a^ All data are expressed as:$\bar{x}\pm sd, n=3$.

^b^ Significance markers: IBM SPSS Statistics 23 software is used for one-way ANOVA test, and then is subjected to post-hoc comparisons with the Waller-Duncan method and a significance level of 0.05.

^c^ Expressed as g·kg^-1^ after multiplying by the factor (0.09) appropriate to lactic acid.
