# Supplementary material for: Analysis of the microbial community structure and flavor components succession during salt‐reducing pickling process of zhacai (preserved mustard tuber)
Source: Food Sci Nutr. 2023 Apr 17;11(6):3154–70. doi: 10.1002/fsn3.3297 (PMC10261794; doi:10.1002/fsn3.3297)
Supplement: Supplementary file 1 — Appendix S1. [file FSN3-11-3154-s001.zip › ═╝║═▒φ/supplementary material 1. Supplementary information of standard reagent and standard curve (monosaccharides).docx]

Supplementary material 1. Supplementary information of standard reagents and standard curves in the detection method of monosaccharides

**Table 1. The information of standard reagents and standard curves**

| Name | Abbreviation of name | CAS No. | Chemical molecular formula | | Peak time（min） | | Slope | | Fitting degree | |
| --- | --- | --- | --- | --- | --- | --- | --- | --- | --- | --- |
| Fucose | Fuc | 99-20-7 | C_12_H_22_O_11_ | | 3.6 | | 0.2313 | | 0.99032 | |
| Rhamnose | Rha | 6155-35-7 | C_6_H_12_O_5_ | | 7.625 | | 0.1509 | | 0.99292 | |
| Arabinose | Ara | 147-81-9 | C_5_H_10_O_5_ | | 8.017 | | 0.258 | | 0.99383 | |
| Galactose | Gal | 381716-33-2 | C_20_H_31_N_3_O_11_ | | 10.184 | | 0.3202 | | 0.99606 | |
| Glucose | Glc | 50-99-7 | C_6_H_12_O_6_ | | 11.742 | | 0.3974 | | 0.99274 | |
| Xylose | Xyl | 58-86-6 | C_5_H_10_O_5_ | | 13.95 | | 0.3613 | | 0.99075 | |
| Mannose | Man | 69-65-8 | C_6_H_14_O_6_ | | 14.709 | | 0.2889 | | 0.99568 | |
| Fructose | Fru | 7660-25-5 | C_6_H_12_O_6_ | | 16.825 | | 0.1234 | | 0.99953 | |
| Ribose | Rib | 50-69-1 | C_5_H_10_O_5_ | | 18.417 | | 0.2304 | | 0.99864 | |
| Galacturonic Acid | Gal-UA | 6037-45-2 | C_18_H_26_O_19_ | | 34.325 | | 0.135 | | 0.99997 | |
| Guluronic Acid | Gul-UA | 15769-56-9 | C_6_H_10_O_7_ | 35.159 | | 0.1483 | | 0.99991 | |  |
| Glucuronic Acid | Glc-UA | 6556-12-3 | C_6_H_10_O_7_ | 36.967 | | 0.238 | | 0.9999 | |  |
| Mannuronic Acid | Man-UA | 6814-36-4 | C_6_H_10_O_7_ | 39.142 | | 0.0967 | | 0.99925 | |  |
