# Supplementary material for: Analysis of the microbial community structure and flavor components succession during salt‐reducing pickling process of zhacai (preserved mustard tuber)
Source: Food Sci Nutr. 2023 Apr 17;11(6):3154–70. doi: 10.1002/fsn3.3297 (PMC10261794; doi:10.1002/fsn3.3297)
Supplement: Supplementary file 1 — Appendix S1. [file FSN3-11-3154-s001.zip › ═╝║═▒φ/supplementary material 2. Supplementary information of standard reagent and standard curve (amino acids).docx]

Supplementary material 2. Supplementary information of standard reagents and standard curves in the detection method of amino acids

**Table 1. The information of standard reagents and standard curves**

| Number | Name | Abbreviation of name | CAS No. | Retention time (min) | Linear equation | Coefficient（r） | Linear range  (ng mL ^-1^) | Limit of quantitation  (ng mL ^-1^) | QC stability  (%) |
| --- | --- | --- | --- | --- | --- | --- | --- | --- | --- |
| 1 | Glycine | Gly | 56-40-6 | 4.38 | y=0.00025x + 0.004274 | 0.9925 | 40~20000 | 40 | 3.05 |
| 2 | L-Alanine | Ala | 56-41-7 | 4.45 | y=0.00159x + 0.006931 | 0.9947 | 10~5000 | 10 | 1.72 |
| 3 | 4-Aminobutyric acid | GABA | 56-12-2 | 4.26 | y=0.001455x + 0.000959 | 0.9948 | 2~1000 | 2 | 2.82 |
| 4 | L-Serine | Ser | 56-45-1 | 4.41 | y=0.003397x + 0.01185 | 0.9915 | 2~500 | 2 | 2.42 |
| 5 | L-Proline | Pro | 147-85-3 | 4.67 | y=0.01493x + 0.01399 | 0.9937 | 1~500 | 1 | 1.85 |
| 6 | L-Valine | Val | 72-18-4 | 5.25 | y=0.01011x + 0.01575 | 0.9922 | 1~1000 | 1 | 2.63 |
| 7 | L-Threonine | Thr | 72-19-5 | 4.49 | y=0.0008978x + 0.002413 | 0.9917 | 4~2000 | 4 | 1.29 |
| 8 | L-Isoleucine | Ile | 73-32-5 | 7.14 | y=0.001686x + 0.001102 | 0.9932 | 2~2000 | 2 | 4.86 |
| 9 | L-Leucine | Leu | 61-90-5 | 7.63 | y=0.01382x + 0.01856 | 0.9957 | 2~1000 | 2 | 2.56 |
| 10 | L-Asparagine | Asn | 70-47-3 | 4.4 | y=7.153e-05x + 0.001332 | 0.9937 | 40~10000 | 40 | 2.11 |
| 11 | L-Ornithine hydrochloride | Orn | 3184-13-2 | 4.02 | y=0.006105x + 0.006086 | 0.9919 | 2~1000 | 2 | 2.36 |
| 12 | L-Aspartic acid | Asp | 56-84-8 | 4.49 | y=0.002583x + 0.006005 | 0.9919 | 4~4000 | 4 | 0.88 |
| 13 | DL-Homocysteine | Hcy | 454-29-5 | 4.96 | y=0.0003146x + 0.001339 | 0.9904 | 20~1000 | 20 | ND |
| 14 | L-Glutamine | Gln | 56-85-9 | 4.42 | y=0.001066x + 0.001991 | 0.9972 | 5~1000 | 5 | 0.59 |
| 15 | L-Lysine | Lys | 56-87-1 | 4.01 | y=0.002767x + 0.002917 | 0.9929 | 1~1000 | 1 | 2.91 |
| 16 | L-Glutamic acid | Glu | 56-86-0 | 4.5 | y=0.00343x + 0.006485 | 0.9949 | 2~2000 | 2 | 2.33 |
| 17 | L-Methionine | Met | 63-68-3 | 5.79 | y=0.001879x - 8.641e-05 | 0.9918 | 2.5~2500 | 2.5 | 3.82 |
| 18 | L-Histidine | His | 71-00-1 | 4.04 | y=0.007102x + 0.00952 | 0.9905 | 2~2000 | 2 | 1.88 |
| 19 | L-Phenylalanine | Phe | 63-91-2 | 9.67 | y=0.01148x + 0.01795 | 0.9929 | 1~1000 | 1 | 8.2 |
| 20 | L-Arginine | Arg | 74-79-3 | 4.05 | y=0.007593x + 0.009192 | 0.9935 | 2~1000 | 2 | 1.59 |
| 21 | L-Tyrosine | Tyr | 60-18-4 | 6.29 | y=0.002138x + 0.001669 | 0.9968 | 2.5~2500 | 2.5 | 4.04 |
| 22 | L-Tryptophan | Trp | 73-22-3 | 11 | y=0.00812x + 0.01097 | 0.9953 | 0.5~500 | 0.5 | 1.84 |
